# Potential Role of the Host-Derived Cell-Wall Binding Domain of Endolysin CD16/50L as a Molecular Anchor in Preservation of Uninfected Clostridioides difficile for New Rounds of Phage Infection

**DOI:** 10.1128/spectrum.02361-21

**Published:** 2022-04-04

**Authors:** Wichuda Phothichaisri, Surang Chankhamhaengdecha, Tavan Janvilisri, Jirayu Nuadthaisong, Tanaporn Phetruen, Robert P. Fagan, Sittinan Chanarat

**Affiliations:** a Department of Biochemistry, Faculty of Science, Mahidol University, Bangkok, Thailand; b Department of Biology, Faculty of Science, Mahidol University, Bangkok, Thailand; c School of Biosciences, Florey Institute, University of Sheffield, Sheffield, United Kingdom; d Laboratory of Molecular Cell Biology, Center for Excellence in Protein and Enzyme Technology, Faculty of Science, Mahidol University, Bangkok, Thailand; University of Pittsburgh School of Medicine

**Keywords:** *Clostridioides difficile*, endolysin, cell-wall binding domain, peptidoglycan, surface layer, bacteria-phage coevolution

## Abstract

Endolysin is a phage-encoded cell-wall hydrolase which degrades the peptidoglycan layer of the bacterial cell wall. The enzyme is often expressed at the late stage of the phage lytic cycle and is required for progeny escape. Endolysins of bacteriophage that infect Gram-positive bacteria often comprises two domains: a peptidoglycan hydrolase and a cell-wall binding domain (CBD). Although the catalytic domain of endolysin is relatively well-studied, the precise role of CBD is ambiguous and remains controversial. Here, we focus on the function of endolysin CBD from a recently isolated Clostridioides difficile phage. We found that the CBD is not required for lytic activity, which is strongly prevented by the surface layer of C. difficile. Intriguingly, hidden Markov model analysis suggested that the endolysin CBD is likely derived from the CWB2 motif of C. difficile cell-wall proteins but possesses a higher binding affinity to bacterial cell-wall polysaccharides. Moreover, the CBD forms a homodimer, formation of which is necessary for interaction with the surface saccharides. Importantly, endolysin diffusion and sequential cytolytic assays showed that CBD of endolysin is required for the enzyme to be anchored to post-lytic cell-wall remnants, suggesting its physiological roles in limiting diffusion of the enzyme, preserving neighboring host cells, and thereby enabling the phage progeny to initiate new rounds of infection. Taken together, this study provides an insight into regulation of endolysin through CBD and can potentially be applied for endolysin treatment against C. difficile infection.

**IMPORTANCE** Endolysin is a peptidoglycan hydrolase encoded in a phage genome. The enzyme is attractive due to its potential use as antibacterial treatment. To utilize endolysin for the therapeutic propose, understanding of the fundamental role of endolysin becomes important. Here, we investigate the function of cell-wall binding domain (CBD) of an endolysin from a C. difficile phage. The domain is homologous to a cell-wall associating module of bacterial cell-wall proteins, likely acquired during phage-host coevolution. The interaction of CBD to bacterial cell walls reduces enzyme diffusion and thereby limits cell lysis of the neighboring bacteria. Our findings indicate that the endolysin is trapped to the cell-wall residuals through CBD and might serve as an advantage for phage replication. Thus, employing a CBD-less endolysin might be a feasible strategy for using endolysin for the treatment of C. difficile infection.

## INTRODUCTION

Rising to dangerously high levels in large parts of the world, antibiotic resistance of microorganisms is one of the biggest threats to global health (1). Among them, Clostridioides difficile, formerly named Clostridium difficile, is a Gram-positive multidrug-resistant bacterium and a leading cause of hospital-acquired diarrheal disease which ranges from mild to severe or fatal conditions (2, 3). It often enters the host body by oral ingestion of dormant spores. Under normal conditions, spore germination and colonization of the bacterium are suppressed by gut flora (4–7). The use of antibiotics, however, causes a major change in the microbiota, thereby outgrowth of C. difficile becomes possible. After germination, the bacterium multiplies and produces toxins that cause inflammation and gastrointestinal mucosal damage, and in some cases pseudomembranous colitis (8, 9). Treatment of C. difficile infection (CDI) includes discontinuing the antibiotic and prescribing a different drug, usually fidaxomicin, vancomycin, or metronidazole (10, 11). The bacterium, however, continues to develop strategies to resist antibiotics, for example, drug inactivation, target modification, and efflux pumps (12). Reduction in drug susceptibility as well as resistance to vancomycin and metronidazole have been reported (13, 14). To make matters worse, relapse occurs in 15% to 35% of CDI patients after successful treatment of the initial infection (15, 16). Alternative therapeutic approaches therefore become critically and urgently required now more than ever.

As natural predators of bacteria, phages have received widespread attention for CDI treatment (17–19). Phage replication occurs in a series of events: adsorption of a viral particle to the host surface, injection of phage genome, phage gene expression, genome replication, viral packaging, and finally a release of progeny phages (20, 21). At the late stage of the replication cycle, the phages typically utilize a holin-endolysin system for progeny release through host cytolysis. Generally, a peptidoglycan (PG)-hydrolyzing endolysin does not possess an intrinsic secretory signal. Thus, expression of holin, a transmembrane protein that transports endolysin through the cytoplasmic membrane of the host cell, is required for the access of the enzyme to its substrate—the bacterial PG layer (22, 23). Current application of phages to CDI treatment involves two approaches: whole-phage therapy and phage-derived antimicrobial enzymes (24–27). To date, the former method faces many challenges, for example, narrow host range, lysogeny, and phage resistance (18, 19, 28, 29). Using phage-derived products, on the other hand, confers a broader host-range activity while leaving commensal species unharmed and does not involve difficulty with lysogeny and phage resistance (14, 26, 27, 30). The latter approach is thus attractive as potential treatment for CDI.

Endolysin is a phage-derived hydrolase causing host cytolysis prior to phage progeny release (22, 31). The enzyme encoded in phages of Gram-positive bacteria often consists of two modules: an enzymatically active domain (EAD) and a cell-wall binding domain (CBD). The EAD module in different phages is classified into three main categories—amidase, endopeptidase, and glycosidase—depending on the type of chemical bond within the PG that it cleaves (32, 33). Although there are many studies suggesting potential use of endolysin for CDI treatment, the enzyme is not utilized as yet. One of the main reasons might be because the role of the CBD module seems controversial. For example, C. difficile phage endolysins CD27L, PlyCD, and CD11 do not require the CBD for their catalytic activity (26, 27, 34), whereas in others, such as CTP1L of Clostridium tyrobutyricum phage or PlyC of Streptococcus phage C1, the CBD is essential (35, 36). It is therefore important to understand the necessity and mechanistic function of the domain prior to knowledge translation into future therapy for CDI.

Here, rather than focusing on the function of the EAD, we address the role of the CBD module of uncharacterized endolysins which are encoded in the genomes of clinically isolated phages. We found that the CBD preferentially interacts with the surface polysaccharides of host cells which lack the surface (S)-layer. Besides, dimerization of CBD is important for the interaction and required for anchoring the enzyme to cell-wall remnants after cytolysis. Intriguingly, computational analyses suggest that CBD of C. difficile phage endolysin is structurally related to cell-wall proteins from the bacterial host and confers an improved cell-wall binding activity. Based on these findings, we speculate that phages use structural mimicry of host proteins to design the domain architecture of endolysin. Indeed, we observed the presence of common cell-wall binding domains between phage endolysins and proteins of respective Gram-positive host bacteria. We finally discuss a model in which phages develop a strategic mechanism for preserving neighboring host cells for new rounds of phage infection by utilizing the CBD of their endolysins.

## RESULTS AND DISCUSSION

### CD16/50L is a phage endolysin capable of hydrolyzing the cell wall of C. difficile.

We previously isolated and characterized temperate phages, all of which infect C. difficile from clinical specimens, belonging to the *Myoviridae* family of the order *Caudovirales* (37). Given that all isolated phages show distinct morphology and pattern of genomic DNA restriction digestion, we found it reasonable to assume that they may encode different endolysins. Among them, we selected ϕHN10, ϕHN16-1, and ϕHN50 for further analyses due to their strong virulence toward C. difficile hosts (37). Endolysin from Gram-positive phage is typically a modular hydrolase comprising two functional domains: the N-terminal enzymatically active domain (EAD) and the C-terminal cell-wall binding domain (CBD) (30, 38–40). Based on DNA sequencing results, all endolysins encoded in the genomes of the three phages contained similar protein domain architecture (Fig. S1A). We found that CD10L, the endolysin of the phage ϕHN10, is closely related to the well-characterized endolysin CD27L (38). On the other hand, CD16-1L and CD50L, encoded in the genome of ϕHN16-1 and ϕHN50, respectively, clustered with three endolysins CD119L, CS74L, and CD11, the functions of which are not fully understood (34, 35, 41). Intriguingly, the CD16-1L and CD50L are identical in DNA and amino acid sequences and are slightly distinct to CD119L, i.e., 96.23% identical at the EAD and 98.9% CBD at the amino acid level (Fig. S1A). Moreover, their CBD region is slightly longer than that of other characterized CBDs; particularly the predicted flexible loop regions seemed to be more extended by at least five amino acids (Fig. S1B). Altogether, due to their uniqueness and relatively unexplored nature, we decided to focus on the CD16-1L/CD50L endolysin, hereafter referred to as CD16/50L.

**FIG 1:**
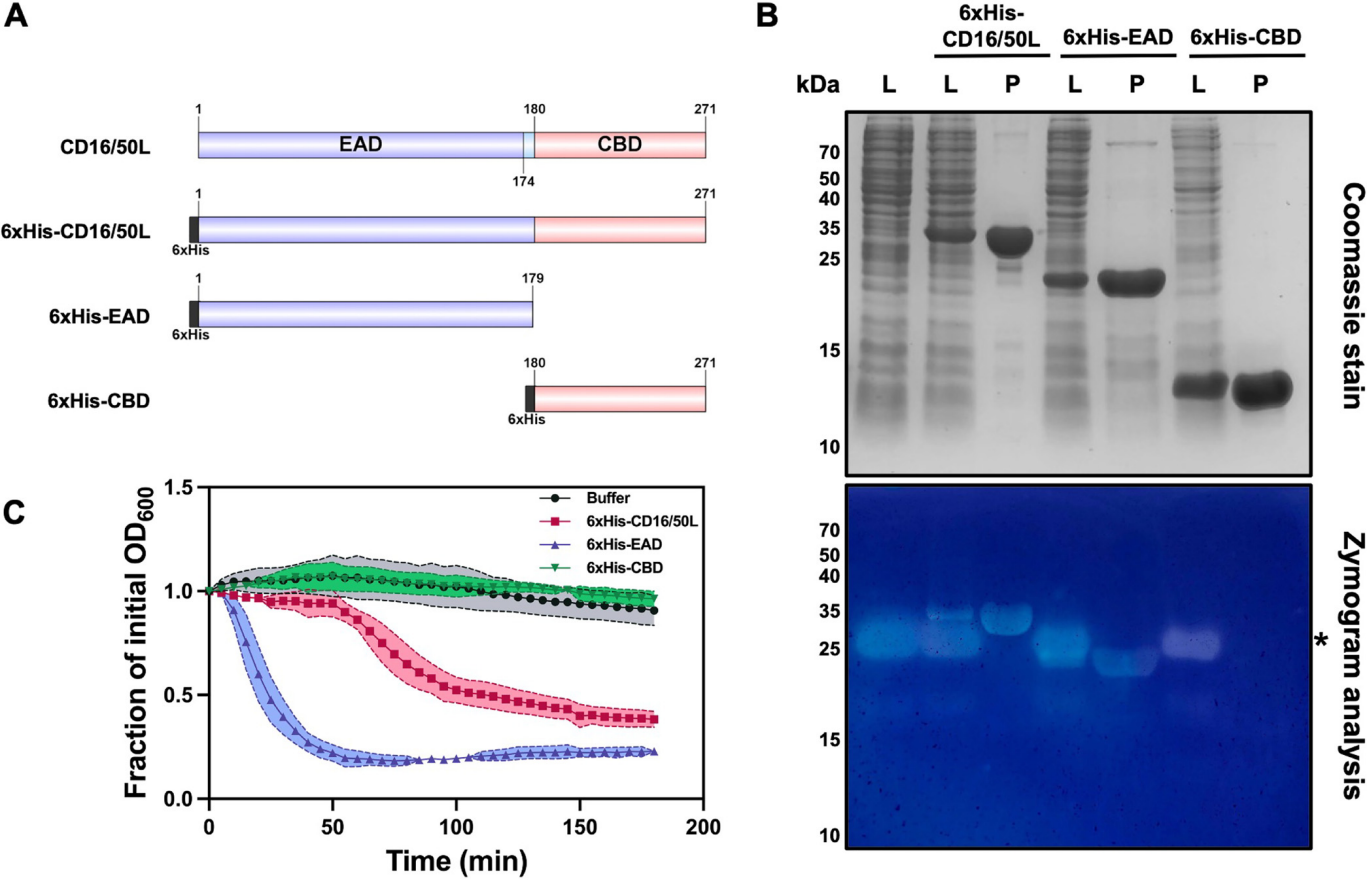
The endolysin CD16/50L is a modular cell-wall hydrolase. (A) Schematic representation of domain structure of endolysin CD16/50L and N-terminally hexahistidine (6xHis)-tagged CD16/50L variants used in this study. CD16/50L is composed of the N-terminal enzymatically active domain (EAD) and the C-terminal cell-wall binding domain (CBD). Numbers indicate amino acid positions in the full-length protein. (B) The EAD of CD16/50L confers a peptidoglycan hydrolase activity. Recombinant protein variants of CD16/50L were expressed in E. coli. Crude lysate (L) and purified proteins (P) were resolved by SDS-PAGE followed by Coomassie blue staining or zymogram analysis using C. difficile peptidoglycan as substrate. Asterisk indicates a false positive band of a highly positively charged protein (see also Fig. S1C; [42]). (C) The full-length and EAD of CD16/50L are able to lyse intact cells of C. difficile. Cytolytic activity of endolysin was assessed by using turbidity reduction assay. Exponentially growing cells were harvested and resuspended in 50 mM Tris-HCl (pH 7.4) in an absence or presence of 2.5 μM purified CD16/50L variants and incubated at 37°C in anaerobic condition for 180 min. The decrement of OD_600_ over time was monitored and plotted. Mean ± SD are shown (*n* = 3).

To confirm whether the predicted domains of CD16/50L are indeed functional EAD and CBD, we recombinantly expressed three N-terminally hexahistidine (6×His)-tagged variants of CD16/50L—the full-length, EAD, and CBD (Fig. 1A)—and tested for the functions. To examine the enzymatic activity, we used an in-gel zymography using cell walls of C. difficile strain HN21, the strain that is the most susceptible to ϕHN16-1 and ϕHN50 infection, as substrate. Indeed, the full-length and EAD variants of CD16/50L conferred strong PG hydrolysis activity while the CBD did not (Fig. 1B). It must be noted that a clear band of approximately 25 kDa was additionally observed in all lanes of the bacterial lysate, even from the untransformed cells (Fig. 1B). The band is most likely not a PG hydrolase, because zymogram control performed without the refolding step also showed the clear band (Fig. S1C). The phenomenon has been reported previously that the zymography is susceptible to false positive results when highly positively charged proteins are assayed (42). Altogether, these results suggest that the CD16/50L EAD is capable of hydrolyzing PG of the C. difficile cells.

Given that C. difficile is a Gram-positive bacterium with multilayered PG cell wall, which is covered by a paracrystalline proteinaceous array of the surface (S)-layer (43, 44), we next asked whether the endolysin CD16/50L is capable of lysing intact C. difficile cells. To this end, we treated exponentially growing HN21 cells with purified recombinant CD16/50L proteins and assayed for cytolytic activity by monitoring the decrement of OD_600_. While buffer control and the CBD of CD16/50L did not show any changes, the full-length and EAD variants decreased the turbidity of cell suspension over time, indicating that the latter two proteins confer catalytic activity against live cells of C. difficile (Fig. 1C). Moreover, in agreement with previous studies of C. difficile phage endolysins, CD16/50L EAD alone seemed to have a higher lytic activity than the full-length protein (Fig. 1C) (26, 30, 34, 40, 45). We therefore conclude that the CD16/50L is a phage-encoded endolysin which is capable of hydrolyzing host PG and of lysing live C. difficile cells.

### The CBD of CD16/50L interacts with surface polysaccharide of C. difficile.

**FIG 2:**
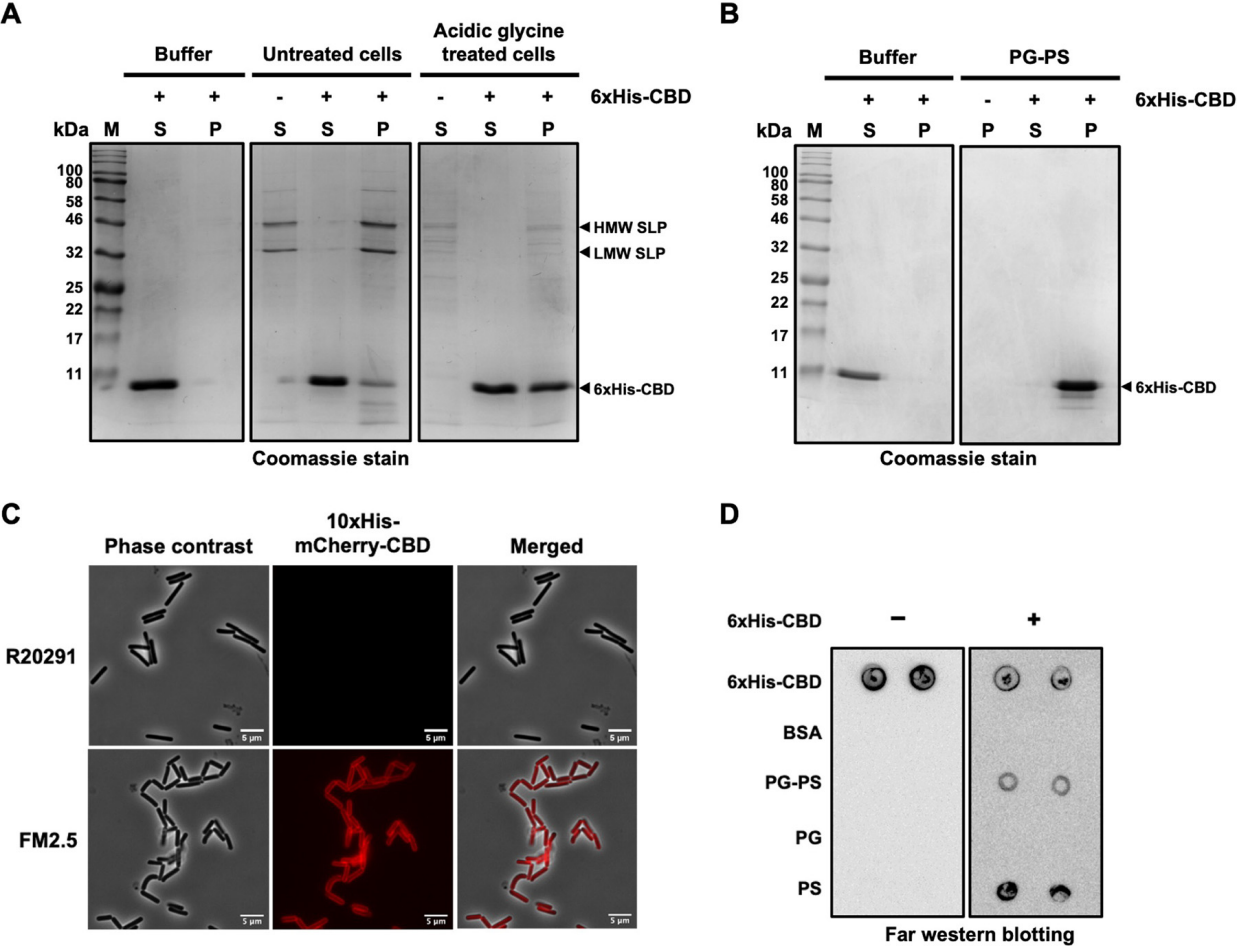
The CBD of CD16/50L interacts with surface polysaccharide of C. difficile. (A) The CBD of CD16/50L binds to cells lacking the surface layer (S-layer) more effectively than the intact cells. Binding of the CBD to whole bacterial cells was investigated using a cell-based pulldown assay. Purified 6xHis-CBD was mixed with buffer, untreated C. difficile, or cells treated with acidic glycine buffer. After incubation and centrifugation, fractions of cell pellet (P) and supernatant (S) were separated in SDS-PAGE, followed by Coomassie blue stain. The high molecular weight (HMW), low molecular weight (LMW) subunits of surface-layer protein (SLP) and the purified 6xHis-CBD are indicated by arrowheads. (B) The CBD of CD16/50L binds to purified peptidoglycan and polysaccharide (PG-PS) complex. Analysis similar to Fig. 2A, but purified PG-PS complex was used. (C) The CD16/50L CBD localizes to the periphery of C. difficile cells deficient in functional S-layer. Affinity purified mCherry-fusion CBD was incubated with exponentially growing wild-type (R20291) or S-layer-deficient (FM2.5) C. difficile cells at 37°C for 30 min. Fluorescence microscopy shows the localization of CBD on the periphery of FM2.5 cells but not that of the R20291. The scale bar represents 5 μm. (D) The CBD of CD16/50L interacts with the secondary polysaccharide of C. difficile cell wall. Purified PG-PS complex, PG, and PS were spotted onto nitrocellulose membrane. Far Western blotting was performed by incubated the membrane with (right panel) or without (left panel) purified 6xHis-CBD in TBS-T containing 2% bovine serum albumin (BSA). Bound CBD was detected by anti-His antibodies followed by HRP-conjugated secondary antibodies and chemiluminescence detection. Spotted 6xHis-CBD and BSA serve as positive and negative controls of far Western blotting assay.

We next asked whether the C-terminal CBD of CD16/50L is capable of interacting with the cell wall of C. difficile. To this end, we used three different *in vitro* assays—cell-based co-precipitation, fluorescence-based detection, and a binding assay using partially purified cell-wall polysaccharides—to study the cell-wall binding activity of the CBD. First, we performed a whole cell co-precipitation assay. As a control, we confirmed that the purified CBD was soluble as the protein alone did not precipitate and was found only in the supernatant after centrifugation at 4,000 × *g* (Fig. 2A; left panel). At this speed of centrifugation, cell pellets of C. difficile could be formed at the bottom of the centrifuge tube, and if resuspended and boiled in standard sample buffer, two apparent bands of the surface layer protein A (SlpA) fragments—the low-molecular-weight (LMW)-SLP and the high-molecular-weight (HMW)-SLP—were observed when analyzed by Coomassie-stained SDS-PAGE (Fig. 2A, middle panel). When we mixed the purified CBD of CD16/50L with C. difficile cells followed by centrifugation, we found that the majority of the recombinant protein was in the supernatant and only a small fraction co-precipitated with the cells (Fig. 2A, middle panel). As it has been reported that CBD of several endolysins binds to the PG layer of the cell wall underneath the compact crystalline 2D lattice of S-layer (46, 47), we hypothesized that the S-layer might block the access of CBD to PG. To test this idea, we treated C. difficile cells with low-pH glycine buffer to extract the surface proteins and again performed the co-precipitation assay. As expected, more CBD of CD16/50L was found in the cell pellet, suggesting that the S-layer might obstruct the interaction of CBD with the peptidoglycan layer (Fig. 2A, right panel). Moreover, partially purified peptidoglycan-polysaccharide (PG-PS) complex, which was extracted from C. difficile with hot SDS followed by DNase I and RNase A treatment, co-precipitated the purified CBD of CD16/50L (Fig. 2B), strongly indicating that the domain indeed binds to the cell-wall polymers of the C. difficile cells.

To extend this finding, we also assessed the cell-wall binding capability of the CBD using a fluorescence-based assay. Because C. difficile cells exhibit a strong autofluorescence at the green wavelength region of emission, red fluorescent protein mCherry was chosen. We fused the CD16/50L CBD with 6×His and mCherry at the N-terminus, expressed in E. coli, and purified by Ni-NTA chromatography. Fluorescence signal of the purified CBD was not observed when it was incubated with the wild-type R20291, a hypervirulent strain of C. difficile (Fig. 2C; top panel). By contrast, when incubating the fusion protein with the R20291-derived FM2.5 strain—an *slpA* insertion frameshift mutant resulting in truncated SlpA protein and thereby the absence of functional S-layer (48)—we found a strong fluorescent signal across the cell surface (Fig. 2C; bottom panel). This result is consistent with the above finding that S-layer might block the access of CBD, and the removal of the S-layer by low-pH glycine buffer permits the interaction between CBD and the underneath PG-PS complex (Fig. 2A). Prompted by these findings, we next asked to which component of PG-PS complex the CBD of CD16/50L binds. The PG-PS complex of C. difficile comprises not only the water-insoluble network of PG—sugar chains with cross-linking peptides—but also water-soluble secondary cell-wall polysaccharides which are covalently attached to the glycan backbone (49). To this end, we fractionated the two portions of insoluble PG backbone and soluble polysaccharides by treating the PG-PS complex with acetic acid followed by a high-speed centrifugation at 15,000 × *g*. Far Western blot analysis showed that the CBD interacted with the water-soluble polysaccharides (PS) rather than to the PG backbone (Fig. 2D). Given that the PS-II is most abundant among several types of PS expressed in most C. difficile ribotypes, we hypothesized that the CBD most likely binds to the PS-II. To test this hypothesis, we again performed a far Western blot analysis and used PG, PS, and PG-PS complex isolated from C. difficile 630, which is known to express only the PS-II type of polysaccharides (44). Indeed, the CBD interacted with the PG-PS complex as well as the PS fraction from this strain (Fig. S2A), suggesting strongly that the domain could bind to the PS-II. Taken together, we conclude that the CBD of CD16/50L binds to the secondary polysaccharides of the PG-PS complex of the C. difficile, which is likely concealed by the S-layer.

### The CBD of CD16/50L forms a homodimer *in vivo* and *in vitro*.

It has been shown previously that CBD of clostridial phage endolysin is a regulatory unit and often functions in a truncated form, which is produced by two mechanisms: CD27L undergoes an autoproteolysis, whereas an in-frame secondary translation occurs in case of both CD27L and CTP1L (38, 39). The CBD fragments can oligomerize and act as a molecular switch to modulate the catalytic activity of the endolysins (38, 39). Intrigued by those findings, we asked whether a similar phenomenon also occurs with CD16/50L. To rule out these possibilities, we first analyzed the sequence of the mRNA encoding CD16/50L, especially around the N-terminus of the CBD region. In contrast to the mRNAs of CD27L and CTP1L which harbor a Shine-Dalgarno sequence followed by an alternative start codon, such a pattern was not observed in the CD16/50L mRNA (Fig. S2B). Also, experimental expression of recombinant CD16/50L protein did not show any band corresponding to a truncated form of CBD (Fig. 1B). From these observations, we infer that CBD of CD16/50L may not function as a free form of truncated protein, unlike those of CD27L and CTP1L endolysins.

**FIG 3:**
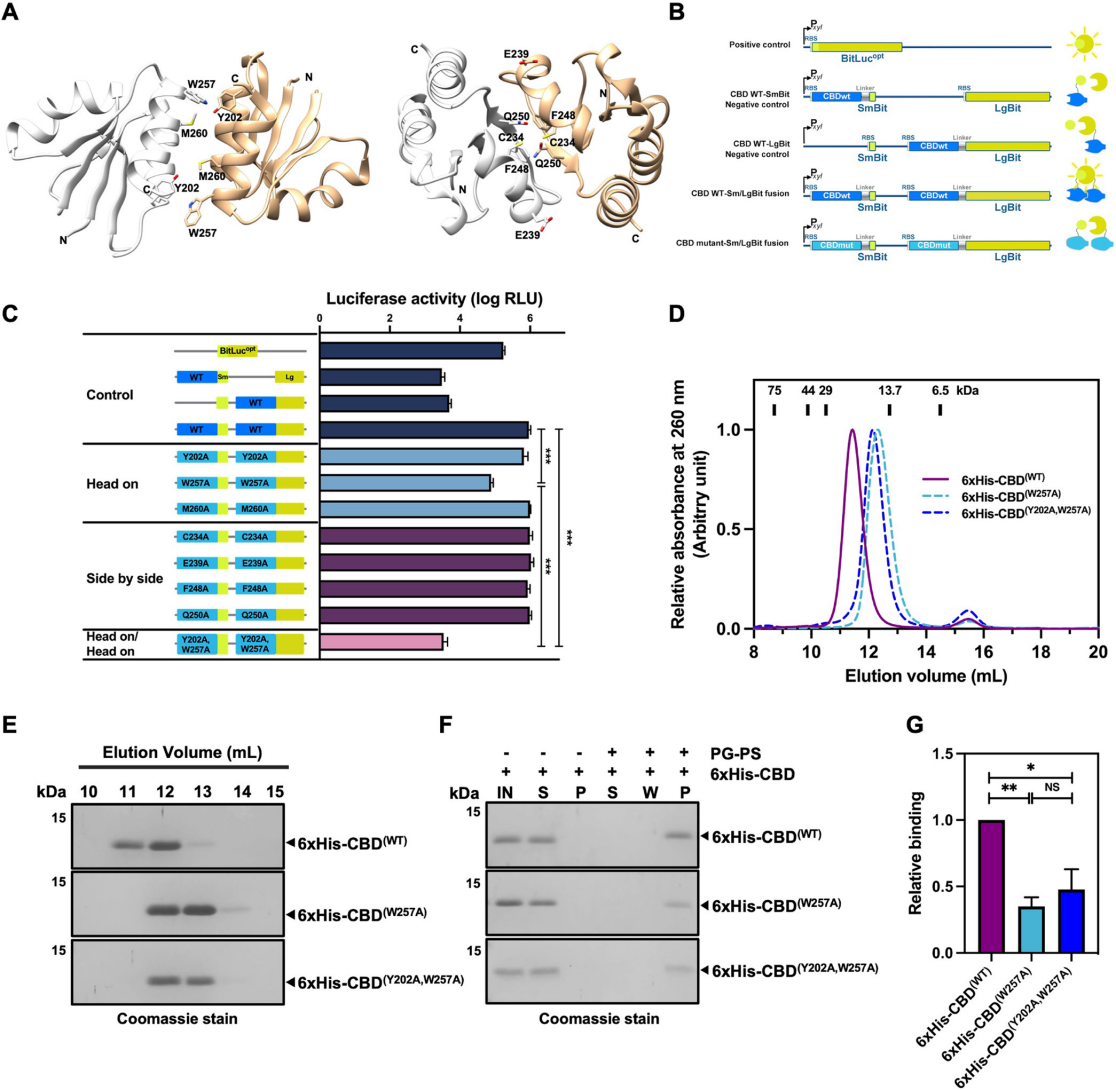
The CBD of CD16/50L forms a homodimer. (A) Modeled structures of CD16/50L’s CBD homodimer predicted by SWISS-MODEL. Ribbon diagram displays the head-on dimer (left) and the side-by-side dimer (right). The interface residues are labeled and shown as a stick model. (B) Scheme of reporter constructs used in a split complementary assay (left panel). Expression of each reporter construct is driven by the xylose-inducible promoter. The full-length luciferase reporter BitLuc^opt^ serves as positive control. Negative controls are the CBD fused with only the large N-terminus (LgBit) or the small C-terminus (SmBit) of BitLuc^opt^. To observe an *in vivo* dimerization, wild-type (WT) or mutant (mut) variants of CBD were fused with each fragment of the reporter. If dimerization occurred, bright luminescent signal could be observed (right panel). Locations of ribosome-binding site (RBS) and linker are shown. (C) CD16/50L’s CBD most likely forms a homodimer *in vivo*. The split complementary luciferase assay using the reporter constructs shown in Fig. 3B was performed. Exponentially growing C. difficile cells harboring each reporter construct were induced with 4% (wt/vol) xylose for 2 h. Furimazine substrate was added and incubated for 5 min. The luminescent signal was then detected and plotted. CBD WT fusions exhibited a strong luciferase activity, while CBD^(W257A)^ and CBD^(Y202A, W257A)^ mutant variants significantly reduced the signal. Mean ± SD are shown (*n* = 3) (***P < 0.001). (D) The CBD of CD16/50L forms a homodimer *in vitro* and the W257 residue is crucial the dimerization. Size exclusion chromatography (SEC; Superdex 75 10/300 GL) analysis of purified 6xHis-CBD protein variants was performed and elution profiles plotted. Elution peaks of protein standards of indicated molecular weight are indicated as black rectangles at the top. (E) SDS-PAGE analysis of the CBD protein variants fractionated by SEC (Fig. 3D). Proteins were separated on SDS-PAGE and detected by Coomassie blue stain. (F) CBD dimerization is most likely crucial for the interaction with the PG-PS complex. Similar to Fig. 2B, but purified CBD mutant variants were analyzed. IN, material before pull-down; S, supernatant; W, wash fraction; P, pellet fraction. (G) Relative binding quantified from Fig. 3F. Protein levels relative to the CBD WT were calculated and plotted. Mean ± SD are shown (*n* = 3) (*P < 0.05, **P < 0.01, NS, not significant).

To gain further insight into the regulatory role of CD16/50L CBD, we addressed the ability of the protein to form an oligomer. It has previously been reported that two dimerization modes of CBD are observed in the crystal of both CD27L and CTP1L endolysins (38). A head-on dimer, a tensed state representing an inactive form of the enzyme, is formed such that the N termini of both monomers are pointing away from the crystallographic dimer interface. On the other hand, a relaxed active state of side-by-side dimer exhibits a more compact configuration, which is prone to aggregation and likely involved in the autocleavage mechanism (Fig. 3A) (38). To address the potential self-interaction, the split-luciferase system which allows us to study protein-protein interactions *in vivo* in the native host C. difficile was used (Fig. 3B) (50). When the full-length enzyme (BitLuc^opt^) was expressed, a bright luciferase signal was observed (Fig. 3C). By contrast, fusion of CD16/50L CBD to either SmBit or LgBit gave rise to a background signal of ∼100-fold lower luciferase activity (Fig. 3C). Strikingly, however, when both luciferase fragments were fused to the CBD and expressed together in C. difficile, the signal was boosted up to 300-fold (Fig. 3C), suggesting that the CBD of CD16/50L is most likely capable of dimerization like other phage CBDs.

Next, we asked whether the CBD of CD16/50L forms a similar head-on or side-by-side dimer, or both, like CD27L and CTP1L. Aiming to obtain candidate interface residues for mutagenesis, we first computationally modeled head-on and side-by-side dimeric structures of the CD16/50L CBD using SWISS-MODEL with those of CD27L as templates (Fig. 3A) (38, 51). We next sought to predict the residues defined by an interface contact distance of 3.5 Angstrom and initially selected seven putative residues for alanine scanning mutagenesis: Y202, W257, and M260 for the head-on configuration, and C234, E239, F248, and Q250 for the side-by-side (Fig. 3A). The luciferase activity of SmBit/LgBit fusion proteins when individual CBD amino acids were replaced by alanine was monitored. Strikingly, we observed that only W257A, a head-on interface mutant variant, showed a reduction in luciferase activity, suggesting that W257 might be the major residue contributing to the dimerization (Fig. 3C). In the predicted dimer, W257 lies adjacent to the aromatic amino acid Y202 of the opposing monomer (Fig. 3A), we thus hypothesized that both aromatic residues may contribute to the stability of the head-on dimer probably via a hydrophobic interaction. To this end, we generated a double point mutant variant (Y202A and W257A) of CD16/50L CBD fusion protein and tested again for the luciferase activity. Indeed, the combination of the two-point mutations resulted in a significant decrease in the luciferase signal, which was even lower than that of W257A single point mutation and was at approximately the same level of the negative controls (Fig. 3C).

Prompted by the *in vivo* finding, we conducted an *in vitro* experiment by expressing and purifying 6×His-tagged CBD variants, and analyzing dimer formation by size exclusion chromatography using an analytical Superdex 75 10/300 GL column. A single peak of purified wild-type CBD (CBD^(WT)^) was eluted in fractions at 12 mL (Fig. 3D to 3E). The position of the peak in the elution profile corresponded to approximately 24 kDa. By contrast, when both CBD^(W257A)^ and CBD^(Y202A, W257A)^ head-on mutant variants were analyzed, the main peaks were slightly shifted compared with the CBD^(WT)^ and eluted at 13 mL, indicating smaller molecules of CBD head-on mutant variants (Fig. 3D to 3E). Assuming that the shape of CBD is not significantly affected, we imply that the two mutations prevent the formation of active head-on dimer, resulting in a monomeric conformation of the CBD. Given that dimerization of CBD may play a role in the interaction with the C. difficile cell wall, we next tested the hypothesis by co-precipitation. Indeed, CBD^(W257A)^ and CBD^(Y202A, W257A)^ head-on mutant variants have a 2-fold lower affinity to the PG-PS complex than CBD^(WT)^ (Fig. 3F to 3G). Taken together, we conclude that the CBD of CD16/50L undergoes dimerization, which enhances the interaction between itself and surface polysaccharides.

In agreement with the notion that several hydrophobic residues at the interface most likely contribute to a stabilization of the CD27L head-on dimer (38), we observed that, besides Y202 and W257, five other hydrophobic residues—V194, I198, L201, M260, and I264—are also located at the interface of the homodimer (Fig. S3A to S3B), suggesting that hydrophobic interaction might be a driving force for dimerization. Although we cannot completely rule out the possibility that a side-by-side dimer might exist, based on the above findings we conclude that the CBD of CD16/50L is able to form a head-on homodimer *in vivo* and *in vitro*.

### CD16/50L EAD hydrolyzes C. difficile and certain clostridial relatives but not distant bacteria.

It has been proposed that CBD dictates the range of host/strain-specific cytolysis by recognizing and noncovalently interacting with specific molecules within the cell envelope, thereby affecting the activity range of the enzyme (52, 53). Also, dimerization of endolysin CBD can mediate a molecular switch that controls the catalytic activity of the EAD (38, 39). Intrigued by these observations and our findings, we speculated that the dimerization of CD16/50L CBD may also regulate functions of the endolysin, namely, host range specificity and cytolytic activity. To this end, we tested the hypothesis by treating a total of 14 C. difficile strains, nine other Gram-positive, and three Gram-negative bacteria with purified CD16/50L harboring or lacking the functional CBD (Fig. 1C and Fig. S4A to S4D, Table 1). All strains remained intact in buffer alone. As expected, CD16/50L was unable to lyse any of the Gram-negative bacteria (Shigella boydii, Enterobacter aerogenes, and Escherichia coli) and non-Clostridia Gram-positive species (Lactobacillus reuteri, Listeria monocytogenes, and Staphylococcus aureus) regardless of the presence or absence of the CBD (Fig. S4A to S4B).

**TABLE 1:** A cytolytic spectrum of CD16/50L

Gram	Bacteria	Strain	PCR ribotype	Source country	Lytic activity^*a*^			Reference
					6xHis- CD16/50L^(WT)^	6xHis- CD16/50L^(W257A)^	6xHis-EAD	
Positive	C. difficile	R20291	027	UK	+	+	+++	N. Minton
Positive	C. difficile	630	012	UK	++	++	+++	N. Minton
Positive	C. difficile	N/A	001	UK	++	++	+++	N. Minton
Positive	C. difficile	N/A	017	UK	++	++	+++	N. Minton
Positive	C. difficile	N/A	020	UK	++	++	+++	N. Minton
Positive	C. difficile	N/A	023	UK	++	++	+++	N. Minton
Positive	C. difficile	N/A	046	UK	+	++	++	N. Minton
Positive	C. difficile	N/A	056	UK	++	++	+++	N. Minton
Positive	C. difficile	N/A	081	UK	++	+++	+++	N. Minton
Positive	C. difficile	N/A	095	UK	+	++	+++	N. Minton
Positive	C. difficile	N/A	106	UK	+	++	+++	N. Minton
Positive	C. difficile	HN21	017	Thailand	++	++	+++	(101)
Positive	C. difficile	RA1	NT1	Thailand	+	++	++	(101)
Positive	C. difficile	RA2	NT2	Thailand	+	++	++	(101)
Positive	Clostridium bifermentans	HN12	N/A	Thailand	++	++	+++	(101)
Positive	*C. bifermentans*	HN15	N/A	Thailand	++	++	+++	(101)
Positive	Clostridium acetobutylicum	ATCC 824	N/A	USA	+++	+++	+++	ATCC
Positive	Clostridium perfringens	ATCC 13124	N/A	USA	−	−	−	ATCC
Positive	C. perfringens	DMST 16637	N/A	Thailand	−	−	−	DMST
Positive	Lactobacillus reuteri	P7	N/A	Thailand	−	−	−	(102)
Positive	L. reuteri	P10	N/A	Thailand	−	−	−	(102)
Positive	Listeria monocytogenes	DMST 23145	N/A	Thailand	−	−	−	DMST
Positive	Staphylococcus aureus	ATCC 25923	N/A	USA	−	−	−	ATCC
Negative^*b*^	Enterobacter aerogenes	DMST 2720	N/A	Thailand	−	−	−	DMST
Negative^*b*^	Shigella boydii	DMST 30245	N/A	Thailand	−	−	−	DMST
Negative^*b*^	Escherichia coli	ATCC 35150	N/A	Thailand	−	−	−	ATCC

a +++, 50% reduction of OD_600_ within 60 min; ++, 50% reduction of OD_600_ within 180 min; +, <50% reduction of OD_600_ after 180 min; −, No observable differences as compared with the control. N/A, not applicable; ATCC, American Type Culture Collection; DMST, Department of Medical Sciences Thailand.

b Gram-negative bacteria were treated in a presence of 0.1 mM EDTA.

Next, we tested the cytolytic activity of the enzyme against different strains of C. difficile and closer relatives in the same class of Clostridia. Surprisingly, CD16/50L EAD was able to hydrolyze not only all C. difficile strains that we tested, but also Clostridium bifermentans (strains HN12 and HN15) and Clostridium acetobutylicum (strain ATCC 824), suggesting that they share a amide bond between the glycan moiety (N-acetylmuramic acid, MurNAc) and the peptide moiety (l-alanine) at the N-terminal of the stem peptide—the substrate of the N-terminal amidase of the endolysin (Fig. S4C and S4D). Intriguingly, all Clostridia species except for Clostridium perfringens (strains ATCC 13124 and DMST 16637) were susceptible to CD16/50L EAD (Fig. S4D). Although the amide bond in the peptidoglycan of all clostridial bacteria is likely invariable, the discrepancy in the cytolytic effect of CD16/50L on different species in the class Clostridia might come from the difference in the N-deacetylation of the MurNAc and/or the *N*-acetylglucosamine (GlcNAc) between different strains of Clostridia (54, 55). Given that more than 90% of the peptidoglycan of C. difficile is N-deacetylated (55), the modification of PG might provide a recognition platform for EAD substrates. Another possible reason could be that C. perfringens contains a unique amino acid composition and arrangement of interpeptide bridge (56). In most cases, the stem peptide in Clostridia consists of L-alanyl-d-glutamyl-meso-diaminopimelyl (DAP)-d-alanyl-d-alanine linked to the lactyl group of MurNAc (54, 57, 58). By contrast, a rare isoglutamine and L,L-DAP, an epsilon-carboxy derivative of lysine, are used in C. perfringens (56). Moreover, the L,L-DAP is cross-linked to the L,L-DAP residue of another peptide chain via a glycine in C. perfringens, while in C. difficile and other relatives meso-DAP is cross-linked directly to the d-alanine of another peptide chain without interpeptide bridge (54, 56–58). Altogether, we conjecture that the difference in post-synthetic modification of the PG backbone and/or amino acid composition of the interpeptide bridge between species may alter peptidoglycan structure, thereby affecting the substrate accessibility and recognition by the endolysin. It is also important to note that, in accordance with the above result (Fig. 1C), CD16/50L EAD always showed a much faster hydrolysis half-life than full-length endolysin in all cases, although hydrolysis of each strain exhibited deviating kinetics (Fig. S4C to S4D). Taken together, we conclude that CD16/50L EAD is able to hydrolyze the cell wall of certain species in the class Clostridia but not of distant species in a CBD-independent manner.

### CD16/50L CBD and its dimerization affect differential and strain-specific cytolytic activity of the endolysin.

Given that CBD binds to secondary cell-wall polysaccharides and negatively influences the cytolytic activity of CD16/50L, an abundance of the surface polymer as well as the substrate accessibility of the enzyme would directly affect the endolysin-dependent cell lysis. Also, it has been shown that different strains of C. difficile contain distinct levels of the surface polysaccharide (49). Moreover, the S-layer cassette, which encodes the key protein component SlpA of the surface layer of C. difficile, not only exhibits a high degree of variability but also undergoes frequent recombination (59, 60). We therefore speculated that the lack of CBD or the dimerization-deficient mutant variant of CD16/50L harboring W257A substitution in the CBD region would enhance the cytolytic activity of the enzyme in a strain-specific manner. Indeed, we observed a difference between CD16/50L^(WT)^ and CD16/50L^(W257A)^ in kinetics of cytolysis against most C. difficile strains: i.e., 001, 017, 020, 056, 081, 095, 106, 046, RA1, and RA2 (Fig. S4C). Intriguingly, there was no or only a minute difference against *C. bifermentans*, C. acetobutylicum, and certain strains of C. difficile, i.e., HN21, R20291, 630, and 023 (Table 1 and Fig. S4C to S4D). It is also important to note that the EAD variant caused a different degree of cytolysis in a strain-specific manner, most likely due to the strain-to-strain variation of the S-layer and cell-wall polysaccharides that prevent accessibility of the enzyme to its substrate, PG. Taken together, we conclude that the CBD and its dimerization—contributing to interaction with the secondary cell-wall polysaccharides—affect the cytolytic function of CD16/50L.

It has been widely proposed that the CBD module of an endolysin confers substrate specificity to the EAD module targeting specific bond within the peptidoglycan layer (58). In fact, several endolysins show a strong dependency on the CBD, the lack of which dramatically reduces or totally abolishes the enzymatic activity (Table S1) (35, 36, 61, 62). By contrast, we found in this study that the CD16/50L EAD did not seem to require the CBD part for the cytolytic activity against C. difficile cells. Furthermore, the CBD could even suppress the lytic activity of the enzyme because the lack of it resulted in an increase in the cytolysis (Fig. 1C and Fig. S4C). As our findings are consistent with other studies performed in several pairs of bacterial cells and endolysins, i.e., in C. difficile, B. anthracis, and L. monocytogenes (Table S1) (26, 63, 64), we indirectly infer that the CBD might have other physiologically beneficial impacts on the endolysins or phage biology besides substrate specificity; otherwise, the modular architecture of the endolysins would not be evolutionarily conserved across diverse bacteriophages (65, 66).

### The CBD of CD16/50L is evolutionarily homologous to the CWB2 domain of C. difficile cell-wall proteins and binds to PG-PS complex more tightly than the host counterpart.

**FIG 4:**
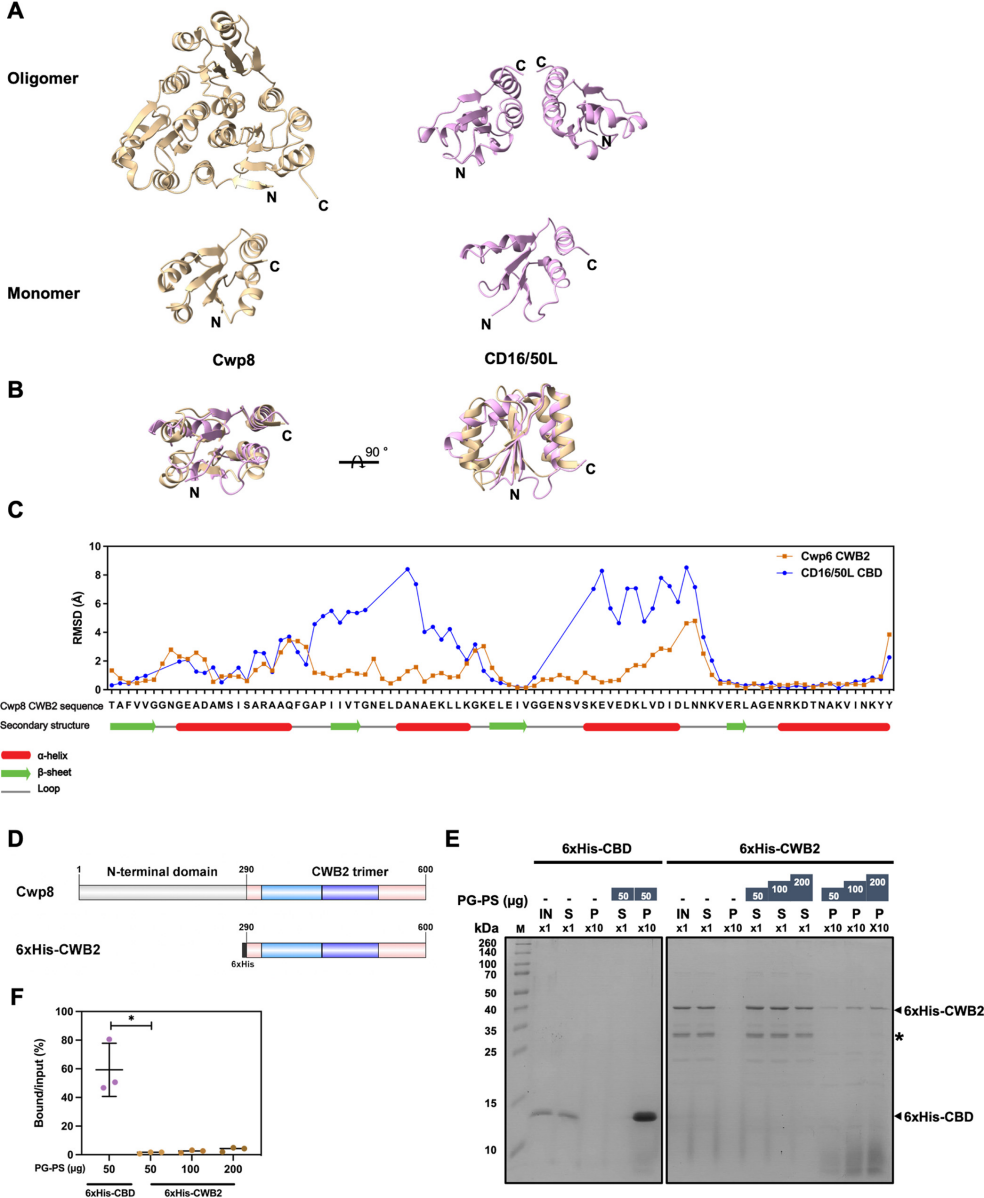
The CBD of CD16/50L is evolutionarily homologous to CWB2 domain of C. difficile cell-wall proteins and binds to PG-PS complex more tightly than the host counterpart. (A) Ribbon diagrams represent the structure of CWB2 (for cell wall binding 2) domain of Cwp8 (PDB: 5J6Q, gold) and CD16/50L’s CBD (similar to Fig. 3A, pink). The top panel shows an oligomer while the bottom panel indicates a monomer. The N- and C-terminus are indicated. (B) Structural superimposition of CWB2 monomer (gold) and the CD16/50L CBD (pink) suggests a remote structural homology. Two views of the structures are related by a 90-degree rotation. (C) Comparison of RMSD of Cα atoms between Cwp8 CWB2:Cwp6 CWB2 (blue) and Cwp8 CWB2:CD16/50L CBD (orange) is shown. The Cwp6 CWB2 serves as a control. The amino acid sequence and secondary structure of the Cwp8 CWB2 are shown. An alpha helix (red), a beta sheet (green), and a loop (gray) are indicated. (D) Scheme of Cwp8 and N-terminally 6xHis-tagged Cwp8 CWB2. The Cwp8 consists of the N-terminal domain and a triangular trimer of CWB2 domain. The number at the start and end of each protein/domain indicates the amino acid position. (E) The CD16/50L CBD binds to PG-PS complex more tightly than the bacterial CWB2 domain. Similar to Fig. 2B but PG-PS binding activity of CBD and CWB2 was compared. An equal volume of 5 μM purified CD16/50L CBD or CWB2 was incubated with indicated amount of PG-PS complex at 37°C for 20 min. After centrifugation and wash, pellets were resuspended in one tenth (×10) of the original volume. Then an equal volume of input (IN), supernatant (S), and pellet (P) were subjected to SDS-PAGE, followed by Coomassie blue stain. The 6xHis-CWB2 and 6xHis-CBD position are marked as arrowhead. The asterisk denotes a degraded fragment of the purified protein. One representative experiment from three biological replicates is shown. (F) Protein band intensity shown in Fig. 4E was quantified, percentage of PG-PS bound protein over the input calculated and plotted. Mean ± SD are shown (*n* = 3) (*P < 0.05).

The CBD of C. difficile phage endolysins is unique in protein sequence and structure, and as yet unassigned to any known protein-domain family in Pfam or InterPro database (67, 68). This could be because many viruses including phages acquire protein and domain sequences via horizontal gene transfer and/or from their hosts, and subsequently often simplify the domain composition and architecture (69). To gain deeper insight into the role of the CBD module, we performed an unbiased approach using HHpred, a computational algorithm for remote protein homology detection that implements pairwise comparison of profile hidden Markov models (HMMs) (70). We reasoned that the detection of a distant CBD homolog would give us a clue to how the domain functions. Strikingly, we found that the domain is remotely homologous to the CWB2 domain (cell-wall binding domain 2, Pfam: PF04122) of C. difficile cell-wall proteins (Table S2). The CWB2 domain is broadly found not only among Actinobacteria and Firmicutes, a phylum to which C. difficile belongs, but also in certain species of archaea and eukaryotes (67). It typically occurs in triple tandem repeats and is a part of cell-wall and surface proteins, including SlpA, and functions as a cell wall-anchoring module that binds to the teichoic acid-like polysaccharide type II (PS-II) on the surface of C. difficile cells (44, 71). Crystal structures of two cell-wall proteins Cwp6 and Cwp8 show that the triple tandem repeats of CWB2 are required for the formation of a triangular pseudotrimer (72). Secondary structure comparison between the modeled CD16/50L CBD and Cwp8 CWB2 revealed that both domains shared an open α-β Rossmann fold with an α-carbon average root-mean-square deviation (RMSD) between pruned atom pairs of 0.827 Å (Fig. 4A to 4C). In essence, CD16/50L CBD is slightly shorter than the Cwp8 CWB2. We observed major differences between the two domains at the flexible loop region flanked by two α-helices, α_1_ and α_2_, and at the α_3_ helix, which was more relaxed and partially unfolded in the case of CD16/50L CBD (Fig. 4B to 4C). Acquisition of host protein sequences is commonly observed in diverse groups of viruses and phages. Often, the host-originated sequences are subsequently adapted and domain compositions simplified (69). This might be the case for C. difficile phages, too. Taken together, we infer that the phages most likely have acquired the protein sequence of CWB2 domain from the hosts, simplified and used it as a part of phage cell-wall hydrolases—endolysins.

Similar to CWB2, CD16/50L CBD was capable of interacting with secondary cell-wall polymer, including PS-II type of polysaccharides (Fig. 2B and D and Fig. S2A) (71). Intrigued by this finding, we next asked whether the adaptation and simplification of CBD would change the saccharide-binding affinity. To this end, we recombinantly expressed in E. coli N-terminally 6×His-tagged CWB2 domain obtained from the *cwp8* gene and compared the activity by using the PG-PS binding assay (Fig. 4D). In agreement with previous findings, we also observed the interaction of 6×His-CWB2 protein with the surface polymer as it was co-precipitated with insoluble PG-PS complex in a dose-dependent manner (Fig. 4E). Strikingly, the CWB2 domain seemed to bind to PG-PS complex with an ∼45-fold lower binding activity than CD16/50L CBD (Fig. 4E and F). Because both CWB2 and CD16/50L CBD interacted with the PG-PS complex, we next asked whether they exhibit a competitive binding. To this end, we first tested by mixing the PG-PS complex with both CWB2 and CBD at various combinations of protein concentration ratio. We observed that when we fixed the concentration of CWB2 and titrated the CBD, intensity of PG-PS bound fraction of CWB2 stayed unchanged while that of CBD increased in a dose dependent manner (Fig. S5A). Likewise, when we fixed the concentration of CBD and titrated the CWB2, similar observation was achieved (Fig. S5B), implying that the interacting molecules of CBD and CWB2 on the bacterial polysaccharides might be different and thereby the competitive binding was not observed.

From the above findings, we also noticed that not only CWB2 was found in the PG-PS bound fraction, but also substantial amount of the protein was in the supernatant, suggesting that the binding might reach equilibrium. Nevertheless, titration of CBD did not alter the balance, again indirectly implying that competitive binding might not happen. By contrast, no CBD was observable in the supernatant, so we took into an account the possibility that the CBD binding may not reach the equilibrium yet, thereby no binding competition—if any—could be detected. To this end, we performed a modified PG-PS binding assay by reducing the amount of the PG-PS complex and pre-binding the polysaccharides with concentrated CBD. We hypothesized that if CWB2 binds to the same site as CBD on the polysaccharides, the titration of CWB2 into PG-PS complex pre-equilibrated with CBD will result in the release of CBD. By contrast, if the two proteins bind to different sites on the PG-PS complex, no pre-bound protein will be released and both proteins will be precipitated together with the PG-PS pellet. Indeed, when we precipitated the PG-PS complex with 6×His-CBD and then treated with different concentrations of purified 6×His-CWB2, we again observed that the amount of PG-PS pellet-bound CBD remained unchanged while the intensity of CWB2 band increased upon titration (Fig. S5C). Taken together, these findings suggest that the interacting sites of CBD and CWB2 on the polysaccharides may be different.

Currently, we cannot rule out the possibility that the surface polysaccharides of C. difficile have a strong binding preference for CWB2 in particular cell-wall proteins. Distinct natural localization of endolysin and cell-wall proteins may also be important to dictate different polysaccharide-binding activities. Once secreted (and processed in some cases such as SlpA) from the cytosol, mature CWB2-domain proteins are localized at the S-layer of C. difficile cells (44, 72). On the other hand, the endolysin CD16/50L is secreted from the cytosol through holin and functions within the CWB2-free layer of peptidoglycan beneath the S-layer (71, 73). In such a case, the competition for the polysaccharide interaction between CBD and CWB2-domain proteins, if any, may be in favor of the endolysin, because CWB2-domain proteins are absent in the peptidoglycan layer when the enzyme emerges from the cytosol. Also, it has been recently shown that the CWB2-containing SlpA, the most abundant S-layer protein of C. difficile, is able to self-assemble into the crystaline array of S-layer (74). Therefore, the higher order lattice of native CWB2-domain proteins might collectively bind to the wall saccharides stronger than the individual subunit. Nevertheless, it is reasonable to assume from our findings that the simplified cell-wall binding module in phage endolysin might mimic the structure of its host counterpart and interact significantly tighter with cell-wall polysaccharides.

### The CBD of CD16/50L anchors the endolysin to bacterial post-lytic remnants and prevents a successive round of cytolysis.

Intrigued by the above findings, we speculated that the simplified yet improved cell-wall binding characteristics of CD16/50L CBD might be an evolutionary adaptive advantage for phage survival. Physiologically, phages of Gram-positive hosts often use a holin-endolysin pathway to release progeny virions during the late stage of the lytic cycle (22, 31). A small protein holin perforates the inner membrane of bacteria and allows endolysin to escape from bacterial cytoplasm to attack the peptidoglycan layer of the hosts. Subsequent endolysin-dependent peptidoglycan disruption and the difference in osmotic pressure between inside and outside of the cells ultimately cause cell rupture, and thus the release of phage progeny (73). However, the CBD-independent catalytic activity of EAD led us to speculate that the CBD portion of endolysin may play physiological roles after the phage lysis. We hypothesized that the endolysin might be captured by the PG-PS complex via its CBD, preventing wider diffusion in the environment. This process is likely beneficial for phages because it would preserve neighboring uninfected host cells for the next round of infection.

**FIG 5:**
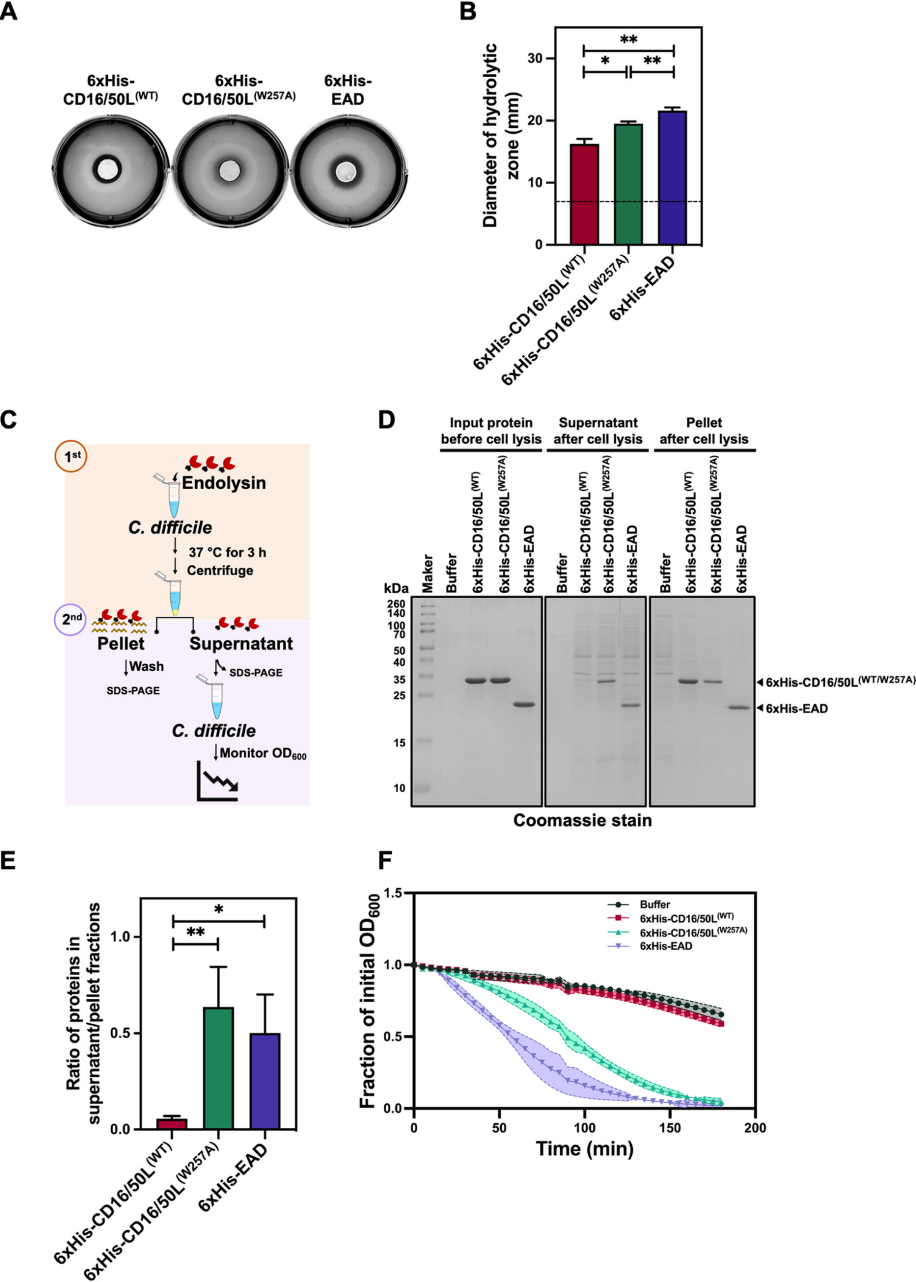
The CBD of CD16/50L anchors the endolysin to bacterial post-lytic remnants and prevents a successive round of cytolysis. (A) The CBD of CD16/50L and its dimerization decrease protein diffusion across a layer of soft agar containing heat-inactivated C. difficile cell debris. A 7-mm diameter hole was created and filled with 100 μM purified CD16/50L protein variants. After incubation at 37°C overnight, diffusion of proteins was assessed by the formation of a lysis halo. One representative experiment from three biological replicates is shown. (B) The diameter of the lytic halo from Fig. 5A was measured and plotted. A dash line indicates the diameter of each hole. Mean ± SD are shown (*n* = 3) (*P < 0.05, **P < 0.01). (C) A diagram depicting a sequential cytolytic assay. The experiment comprises two steps of bacterial cell lysis. Firstly, exponentially growing cells are harvested and resuspended in buffer containing 5 μM purified CD16/50L variants. The mixtures are then incubated at 37°C in anaerobic conditions for 3 h to let endolysin-mediated cytolysis occur. A soluble and insoluble fraction are separated by centrifugation and analyzed by SDS-PAGE followed by Coomassie blue stain. Subsequently, unanchored proteins of soluble fraction are mixed with fresh growing cells, reduction of OD_600_ over time is followed and plotted. (D) The CBD of CD16/50L and its dimerization facilitate the anchoring of the enzyme to cell remnants after endolysin-mediated cytolysis. SDS-PAGE analysis of the CD16/50L protein variants fractionated by centrifugation after the first cytolysis (Fig. 5C). Proteins were separated on SDS-PAGE and detected by Coomassie blue stain. (E) Ratio of soluble and insoluble fraction was quantified from Fig. 5D and plotted. Mean ± SD are shown (*n* = 3) (*P < 0.05, **P < 0.01). (F) Unanchored CD16/50L is able to perform a successive cytolysis of C. difficile cells *in vitro*. Similar to Fig. 1C, but post-centrifuged supernatants after the first cytolysis were analyzed. Mean ± SD are shown (*n* = 3).

To test the hypothesis, we first assayed the diffusion rate of recombinant CD16/50L protein variants across the matrix of soft agar containing heat-inactivated C. difficile debris. After filling a 7-mm-diameter hole with an equimolar amount of purified proteins and incubating overnight at 37°C, we observed that the CBD-less variant diffused significantly faster than the full-length proteins (Fig. 5A and B). Also, the CD16/50L^(W257A)^ variant had a higher rate of diffusion than the CD16/50L^(WT)^. From this result, we infer that the dimerization of CBD might enhance the endolysin capture to the surface polysaccharide trap.

Next, we performed a sequential two-step bacterial lysis assay mimicking the physiological situation of phage progeny (and endolysin) release and monitoring the fate of endolysin after primary cell lysis. (Fig. 5C). First, C. difficile cells were incubated with purified CD16/50L variants at 37°C for 3 h to complete endolysin-dependent cytolysis. PG and cell remnants were then separated from the aqueous phase by centrifugation and protein content determined (Fig. 5D). In agreement with the purified PG-PS co-precipitation assay, the majority of CD16/50L^(WT)^ was in the PG/cell-remnant-bound pellet, while a vast amount of CD16/50L^(W257A)^ and EAD was found in the PG/cell-remnant-unbound supernatant (Fig. 5D and E), suggesting that the enzyme might be trappable with the remaining post-lytic polysaccharides via the dimerization-competent CBD. We next asked whether the unanchored proteins might be able to start a second round of cell lysis. Indeed, the supernatant of CD16/50L^(W257A)^ and EAD samples still maintained a high cytolytic activity against a new batch of C. difficile cells (Fig. 5F). We thus conclude that the CD16/50L CBD most likely functions as an anchoring domain of the endolysin to post-lytic remnants, helping prevent a successive unnecessary round of cytolysis and thereby increasing availability of host cells for infection by released phage progeny.

Progeny release is tightly coupled with the endolysin-dependent phage lysis. Diffusion of the endolysin may potentially be harmful for neighboring uninfected cells which are potential host of the released phages. It is possible that the enzyme may actively be anchored to the remnant of the infected hosts via its CBD; the CBD-mediated trapping system might be a mechanism to prevent unwanted loss of future host cells and consequently beneficial for phage expansion.

This endolysin-trapping mechanism, mediated by the host-derived CBD module, might be a common strategy among phages of Gram-positive bacteria because a large number of endolysins of these phages not only are modular enzymes containing at least one cell-wall associating module, but the module is also closely or remotely homologous to a cell-wall binding domain of the phage’s respective hosts (Table S3) (75–77). Phages of Gram-negative bacteria, on the other hand, may use a different approach as they often lack a cell-wall binding domain in their endolysins (78, 79). We speculate that the CBD-mediated endolysin trapping mechanism may not suit in Gram-negative environment due to two plausible reasons. First, Gram-negative bacteria have a much thinner peptidoglycan layer and lower amount of secondary polysaccharides (80), which are required for trapping endolysin via CBD. Second, spanin-mediated inner membrane-outer membrane (IM-OM) fusion during phage lysis might be able to capture endolysins in a membrane-bound vesicle (81, 82). In any case, the endolysin-trapping mechanism is possibly beneficial for phage expansion because it helps secure uninfected hosts for progeny phages, especially in a biofilm environment where bacterial hosts are in close proximity to one another.

### Conclusion.

Endolysin is a PG hydrolase required for phage lysis at the late step of the lytic cycle. The enzyme encoded in the genome of phages of Gram-positive bacteria usually consists of two functional domains: EAD and CBD. It is commonly accepted that the EAD is a catalytic module hydrolyzing the peptidoglycan of host cells. CBD, on the other hand, has a contradictory function; CBDs of certain endolysins are absolutely required for catalytic activity, while others are not (63, 64). Although individual endolysins may require a unique regulation, the necessity and mechanistic function of the domain may also lie in other aspects of endolysin in phage biology.

Here, we show that CBD of an uncharacterized endolysin CD16/50L interacts with the surface polysaccharide of host cells, especially cells that lack the S-layer. The domain is able to form a homodimer, which is required for optimal polysaccharide interaction. The CBD seems to be derived from the host genome, with high structural similarity to the CWB2 domain commonly found in C. difficile cell-wall proteins. Notably, the simplified phage domain exhibits superior cell-wall binding activity to its host counterpart. Although CBD is not required for CD16/50L catalytic activity, it prevents the diffusion of the enzyme into the environment by anchoring itself to the post-lytic insoluble cell-wall remnants. As a result, the endolysin trapping mechanism most likely protects neighboring uninfected cells which are potential hosts of the released progeny phages.

Phages use endolysin merely as a stepping stone to a successful phage expansion; they intend not to kill bacterial hosts more than necessary. On the contrary, for CDI treatment our intention is to put an end to all the pathogens, therefore we may need to strategize differently to the phages. Our findings indicate that while the presence of its CBD might interfere with cell lysis, the EAD of CD16/50L confers a cytolytic activity against a broad spectrum of C. difficile strains. Moreover, the content of surface polysaccharides as well as the integrity of S-layer seem to affect the endolysin activity. It is therefore important to determine to what extent these two cell-wall components guard the bacterial cells against endolysin-dependent cell lysis. Although there are still many challenges ahead, it is tempting to speculate that a combination of removal of the PG guardians and the usage of CBD-less endolysin might contribute to a step toward endolysin treatment for CDI in the future.

## MATERIALS AND METHODS

### Bioinformatics analysis.

Multiple alignments of the endolysin sequences including CD27L (YP_002290910.1), CDC2L (YP_001110754.1), CD119L (YP_529586.1), CD11 (WP_009895119.1), PlyCD (WP_003435466.1), CTP1L (YP_003856822.1), CS74L (YP_007237262.1), CD10L (OK557798), CD16-1L (OK557799), and CD50L (OK557800) were performed using Clustal Omega (version 1.2.4) (83) with default settings. Jalview (version 2.11.1.4) (84) was used to visualize the multiple alignment results. Remote homology of cell-wall protein was analyzed using hidden Markov models (HMMs) as implemented in HHpred server (70). Bacterial proteins and phage endolysins were retrieved and analyzed using protein databases including RCSB PDB (85), Pfam (67), and Uniprot (86).

### Structural modeling.

The dimeric structure of CD16/50L CBD was predicted by SWISS-MODEL online server (51) using CD27L (PDB: 4CU5) as template. The structures of other CBDs from C. difficile phage endolysins were predicted using AlphaFold (87). Structural comparison was carried out using UCSF ChimeraX Daily Build version (version 1.3; 2021-09-07) (88).

### Bacterial strains and growth conditions.

All bacterial strains and plasmids used in this study are indicated in Table S4 and S5, respectively. E. coli strains were routinely grown on Luria-Bertani (LB) agar or LB broth (Himedia) supplemented with 100 μg/mL Ampicillin, 15 or 30 μg/mL chloramphenicol, and 50 μg/mL kanamycin when appropriate, grown aerobically at 37°C. Plasmids were transformed using standard protocol (89) and maintained in E. coli XL10-Gold. E. coli strains Rosetta (DE3) and BL21(DE3) were used for protein expression. E. coli CA434 (HB101 carrying R702) was used for conjugation of a plasmid from E. coli into C. difficile strain R20291 (90). All strains were stored at −80°C in 20% glycerol.

C. difficile strains were routinely grown on brain heart infusion (BHI) agar (Himedia) or TY broth (3% tryptose, 2% yeast extract) (Himedia) in an anaerobic cabinet (Don Whitley Scientific), at 37°C, in an atmosphere of 10% CO_2_, 10% H_2,_ and 80% N_2_. All media were pre-reduced overnight in the anaerobic workstation before inoculation. Thiamphenicol (15 μg/mL) and colistin (50 μg/mL) were added to the media where appropriate. Four percent (wt/vol) final concentration of d-xylose (Merck) was used for induction of the P_xyl_ promoter in the C. difficile expression vectors described below.

### Construction of expression and reporter plasmids.

All plasmids and oligonucleotides used in this study are outlined in Table S5 and S6, respectively. To construct expression vectors for 6×His-CD16/50L full-length, 6×His-EAD, and 6×His-CBD, the genes were amplified by PCR from phage ɸHN50 genomic DNA using primer sets (Table S6) as followed: *CD16/50_full-length_* forward/*CD16/50_full-length_* reverse, *CD16/50_full-length_* forward/*CD16/50_EAD_* reverse and *CD16/50_CBD_* forward/*CD16/50_full-length_* reverse, respectively. The genes were placed downstream of hexahistidine (6×His) tag at the NdeI and *Xho*l restriction sites of the pET15b under the control of T7 promoter. The plasmid was transformed into competent E. coli XL10-Gold cloning host. The constructed plasmids were confirmed by Sanger sequencing and subsequently transformed into E. coli BL21(DE3) expression host.

To construct an expression vector for CWB2, an *in vivo* assembly method was performed as previously described (90, 91). Briefly, the CWB2 of *cwp8* was amplified by PCR from C. difficile 630 genomic DNA (GenBank accession no. AM180355.1) using primers (Table S6) *cwp8*_290-600_ forward and *cwp8_290-600_* reverse. The pET15b backbone was amplified using the primers pET15b forward and pET15b reverse. The two primer sets contain homology arm regions, allowing *in vivo* recombination following transformation of linear fragments. The CWB2 region was inserted downstream of 6×His tag under the control of T7 promoter for further expression and purification. To eliminate parental plasmid templates, the pET15b PCR product was treated with DpnI (NEB) as manufacturer’s instruction. The treated PCR mixture was transformed into competent E. coli XL10-Gold cloning host. The constructed plasmids were confirmed by Sanger sequencing and subsequently transformed into E. coli Rosetta (DE3) for expression.

To construct an expression vector for mCherry-CBD fusion, the fused gene was synthesized (Genewiz) and cloned into pET28b downstream of 10xHis tag under the control of T7 promoter. The plasmid was transformed into competent E. coli NEB 5*α* cloning host. The constructed plasmids were transformed into E. coli Rosetta (DE3) expression host.

To construct CBD-BitLuc^opt^ fusion vectors, the split complementary luciferase reporter for C. difficile used in this study was based on BitLuc^opt^ (92), a modified version of a codon optimized luciferase gene, sLuc^opt^ and the NanoBit system (Promega) (50, 93). The anhydrotetracycline-inducible promoter (P_tet_) of BitLuc^opt^ constructs pAF256, pAF257 and pAP118 were replaced by a xylose-inducible promoter (P_xyl_). To generate negative controls including just CBD-SmBit or CBD-LgBit, the CBD was amplified by PCR using primers *CBD-smbit* forward and *CBD-smbit* reverse and cloned into SacI/XhoI-digested P_xyl_-modified pAF256 yielding pWP001 (Addgene: 178596). To construct CBD-LgBit, the CBD was also generated using primers *CBD-lgbit* forward and *CBD-lgbit* reverse and cloned into *Pvu*l/NotI-digested P_xyl_-modified pAF257, producing pWP003 (Addgene: 178598). To produce CBD-SmBit/LgBit, a two-step cloning was performed. The CBD was first amplified by both mentioned primer sets. The PCR product from *CBD-lgbit* primers was cloned into the *Pvu*l/NotI-digested P_xyl_-modified pAP118, yielding pWP002. Subsequently, the PCR product from *CBD-smbit* primers was cloned into SacI/XhoI-digested pWP002, generating pWP004 (Addgene: 178599). pJAK175 (Addgene: 178601), encoding a xylose inducible full-length BitLuc^opt^ was included as positive control.

To create the CBD mutant variants used in the split complementary luciferase assay, alanine substitution of amino acids interface of CBD dimer including Y202, C234, E239, F248, Q250, W257, and M260 were performed using site-directed mutagenesis, according to the Q5 site-directed mutagenesis protocol (NEB). The CBD mutant-SmBit/LgBit was constructed as mentioned above using P_xyl_-modified pAP118 as backbone plasmid. All constructed plasmids were verified by Sanger sequencing, transformed into competent E. coli CA434, and conjugated into C. difficile R20291 (94).

### Protein expression and purification.

Recombinant proteins were expressed in E. coli and purified using Ni-NTA affinity resin (Amintra), according to manufacturer’s instructions. In brief, cultures were grown in LB broth supplemented with 100 μg/mL ampicillin and 30 μg/mL chloramphenicol at 37°C until to OD_600_ reached 0.6. The culture was subsequently induced with 1 mM isopropyl β- D-1-thiogalactopyranoside (IPTG) overnight at 25°C. The cells were harvested by centrifugation at 4,000 × *g* for 15 min at 4°C and stored at −80°C until used. Cells were resuspended in binding buffer A (50 mM NaH_2_PO_4_ (pH 8.0), 300 mM NaCl, 20 mM imidazole and 5% glycerol) supplemented with 50 μg/mL of DNase A. The resuspended cells were lysed by sonication with 10-s on and 30-s off for 15 min at 40% amplitude. The soluble fraction was recovered by centrifugation at 15,000 × *g* for 20 min. The lysate was incubated with pre-equilibrated Ni-NTA resin for 1 h and washed with buffer A. The purified protein was eluted at the 250 mM imidazole containing buffer (50 mM NaH_2_PO_4_ (pH 8.0), 300 mM NaCl, 250 mM imidazole and 5% glycerol) and dialyzed against 50 mM Tris-HCl (pH 7.4), 300 mM NaCl, and 5% glycerol with a dilution factor of 100,000. The dialyzed protein was centrifuged at 20,000 × *g* for 20 min, filter sterilized and stored at −80°C.

### Zymogram assay.

Zymogram analysis was performed according to previously described with modification (95). Briefly, 20 micrograms of purified 6×His-CD16/50L, 6×His-EAD, and 6×His-CBD were separated into 15% sodium dodecyl sulfate polyacrylamide gel electrophoresis (SDS-PAGE) in an absence or presence of 0.2% (wt/vol) autoclave-inactivated C. difficile HN21 strain as a substrate. After electrophoresis, the substrate-free gel was stained with Coomassie blue (InstantBlue). At the same time, the gels containing C. difficile cells were washed twice in distilled water and subsequently incubated with or without renaturation buffer (25 mM phosphate buffer (pH 8.0) and 1% Triton X-100) at 37°C for 16 h. To detect PG hydrolase activity, the gels were washed once with distilled water and then stained for 2 h with 0.1% (wt/vol) methylene blue in 0.01% (wt/vol) KOH. After washing the gel with distilled water, the PG hydrolase activity was observed as a clear zone.

### Cytolytic assay.

Exponential growing cells were harvested by centrifugation at 4,000 × *g* for 10 min. Cells were washed and resuspended in 50 mM Tris-HCl (pH 7.4), and OD_600_ adjusted to 1. Gram-negative bacteria were treated with 0.1 M EDTA for 5 min at room temperature as described previously (96). Cells were centrifuged and washed three times in 50 mM Tris-HCl (pH 7.4). Then 100 μL of 2.5 μM CD16/50L variants or buffer were mixed with 100 μL of prepared C. difficile in a 96-well plate. The reduction of OD_600_ was measured every 5 min for 180 min using microplate reader (Biotek).

### Isolation of cell-wall polymer.

Isolation of surface PG-PS complex was carried out as previously described (44). Briefly, C. difficile R20291 or 630 was grown overnight in TY, subcultured to an OD_600_ of 0.05 in fresh pre-reduced TY broth and incubated in anaerobic condition. Exponentially growing cells were harvested, washed with PBS, and stored overnight at −80°C. Cell pellets were resuspended in 4% (wt/vol) SDS and boiled for 30 min, and cooled to room temperature. The suspension was then centrifuged at 9,000 × *g* for 20 min at room temperature and washed with Milli-Q H_2_O. The washing step was repeated at least three times to remove the residual SDS. The remaining contaminants were eliminated by treating the pellets with DNase I (1 mg/mL) and RNase A (5 mg/mL) in the presence of 20 mM MgSO_4_ at 37°C for 2 h, then by Pronase (2 mg/mL) in 10 mM Tris-HCl (pH 7.4) at 60°C overnight. The solution was mixed with 8% (wt/vol) final concentration of SDS and boiled for 30 min. The sample was centrifuged and washed as previously to remove the residual SDS. The remaining pellet at this stage was resuspended in Milli-Q H_2_O and used as PG-PS complex.

To purify PG, the prepared PG-PS complex was treated with 1 M HCl for 4 h at 37°C to remove covalently cross-linked polysaccharide (97). Then, the insoluble PG portion was harvested by centrifugation at 15,000 × *g* and washed with Milli-Q H_2_O until pH became neutral. The PG was then lyophilized, dry weight measured, and the pellet resuspended Milli-Q H_2_O at a concentration of 5 mg/mL.

To purify PS, the prepared PG-PS complex was treated with 1% (vol/vol) acetic acid at 95°C for 1 h. The soluble PS was recovered by centrifugation at 15,000 × *g*, and dialyzed against Milli-Q H_2_O using dialysis bag with molecular weight cut-offs of 1 kDa (Spectrum). The PS concentration was measured using neutral sugar assay (98).

### Whole-cell binding.

A cell-based pulldown assay was carried out as described previously with slight modification (34, 43). An equal volume of C. difficile R20291 strain was treated with or without acidic buffer containing 0.2 M glycine (pH 2.2) at room temperature for 20 min, recovered and washed twice with Tris-HCl (pH 7.4), and then incubated with 5 μM final concentration of purified 6×His-CBD variants at 37°C for 20 min. The mixtures were centrifuged at 4,000 × *g* for 2 min at room temperature and washed twice with 50 mM Tris-HCl (pH 7.4). Binding of the CBD to bacterial cells was detected using SDS-PAGE, followed by Coomassie blue staining (InstantBlue).

### PG-PS binding assay.

Cell-wall polymer-based pulldown assay was performed as previously described with modification (34). Equal volume of PG-PS dissolved in Milli-Q H_2_O was incubated with 5 μM final concentration of purified 6×His-CBD variants at 37°C for 20 min. The mixtures were centrifuged at 12,000 × *g* for 5 min at room temperature. The pellets were washed twice with 50 mM Tris-HCl (pH 7.4). Binding of the CBD to bacterial cell was detected using SDS-PAGE, followed by Coomassie blue staining (InstantBlue).

### Competitive binding assays.

The competitive binding assays were performed as previously described with modifications (34). For a competitive co-incubation assay, 50 μL of 1 mg/mL purified PG-PS complex was incubated with 5 μM 6×His-CBD and three different concentrations (5, 10, and 20 μM) of 6×His-CWB2, and vice versa. The mixtures were incubated at 37°C for 20 min with agitation rate of 200 rpm. To separate PG-PS bound and unbound proteins, the mixtures were centrifuged at 12,000 × *g* for 10 min at room temperature. The pellets were washed twice with 50 mM Tris-HCl (pH 7.4). The competitive binding between 6×His-CBD and 6×His-CWB2 to PG-PS complex was evaluated using SDS-PAGE, followed by Coomassie blue staining (InstantBlue).

For a competitive displacement assay, 10 μL of 1 mg/mL PG-PS complex was pre-bound to 50 μL of 20 μM purified 6×His-CBD and incubated at 37°C for 20 min with agitation rate of 200 rpm. The mixtures were centrifuged at 12,000 × *g* for 10 min at room temperature. After washing once with 50 mM Tris-HCl (pH 7.4), the pellets were treated with 100 μL of 5, 10, and 20 μM 6×His-CWB2 at 37°C for 20 min with agitation rate of 200 rpm. For the control experiment, an equal volume of 20 μM 6×His-CWB2 was heat-treated at 98°C for 15 min and used to treat the PG-PS pellet. The mixtures were centrifuged at 12,000 × *g* for 10 min at room temperature. The pellets were washed twice with 50 mM Tris-HCl (pH 7.4). The supernatant and pellet fractions were analyzed using SDS-PAGE, followed by Coomassie blue staining (InstantBlue).

### Whole-cell binding and fluorescence microscopy.

One μM final concentration of purified protein was incubated with exponentially growing C. difficile R20291 and FM2.5 (S-layer mutant variant) cells at 37°C for 30 min in anaerobic condition. Then, 120 μL of fixation cocktail containing 20 μL of 1 M NaPO_4_ buffer (pH 7.4), 100 μL of 16% paraformaldehyde, and 4 μL of 25% glutaraldehyde was added and incubated for 30 min. The bacterial cells were centrifuged at 4,000 × *g* for 2 min and washed three times with Tris-buffered saline (TBS, 50 mM Tris-HCl (pH 7.4), 150 mM NaCl). The bacterial pellet was resuspended in 30 μL TBS. Five μL of bacterial cells were mounted onto glass slides. The interaction of the mCherry-CBD fusion was investigated by epifluorescence microscopy on a Nikon Eclipse Ti microscope.

### Far immunoblotting assay.

The binding of protein to carbohydrate component of C. difficile cell wall was performed as previously described (44). Briefly, 5 μL of 5 mg/mL of PG-PS, PG, and PS samples as well as 5 μM 6×His-CBD and bovine serum albumin (BSA) were spotted onto a nitrocellulose membrane (Bio-Rad) and dried for 30 min. Non-specific binding was blocked in 2% (wt/vol) BSA in Tris buffered saline with Tween 20 (TBST) (25 mM Tris-HCl (pH 7.4), 150 mM NaCl, 0.05% Tween 20) at room temperature for 1 h. The membrane was pre-equilibrated in 50 mM Tris-HCl (pH 7.4) at room temperature for 20 min and subsequently incubated with or without 6×His-CBD at room temperature with agitation for 1 h. Membranes were washed with TBST buffer at room temperature for 30 min before incubated with 1:1,000 rabbit anti-His antibody (Cell signaling) at room temperature overnight. Anti-rabbit IgG conjugated to HRP antibody, 1:500, (Cell signaling) was applied and incubated for 1 h, followed by adding of ECL substrate (Bio-Rad). The signal was detected using gel imaging instrument (Syngene G: Box F3 gel Documentation System; New England BioGroup [G:Box F3]).

### Split luciferase complementation.

The C. difficile complementation assay was carried out as described previously with modification (92, 99). Briefly, C. difficile cells carrying CBD-BitLuc^opt^ construct were grown until OD_600_ reached 0.4 to 0.5. The culture was induced with 4% (wt/vol) d-xylose for 2 h. To measure luciferase activity, 50 μL Nano-Glo Luciferase (Promega N1110) was mixed with 50 μL of OD_600_ 0.2 normalized C. difficile culture, according to the manufacturer’s instructions. Measurements were performed in triplicate in a 96-well white flat-bottom plate (Greiner bio-one 655073). The luminescence signal was measured using a Hidex sense microplate reader. Statistical analysis was performed by using Prism 9 (GraphPad, Inc, La Jolla, CA). Statistical significance was calculated using Student’s *t* tests.

### Size exclusion chromatography.

The molecular weight (MW) of the CBD WT and the CBD mutants were estimated by size exclusion chromatography (SEC) using an Äkta purifier CPC10 instrument (GE Healthcare). Approximately 2.5 mg/mL of purified 6×His-CBD^(WT)^ and 6×His-CBD^(W257A)^, and 6×His-CBD^(Y202A, W257A)^ were separately injected onto a Superdex 75 10/300 GL column equilibrated in 50 mM Tris-HCl (pH 8.0) and 500 mM NaCl at a flow rate of 0.5 mL/min. The MW of each species eluting from the SEC column was assessed using the calibration plot of standard proteins: thyroglobulin (669 kDa), conalbumin (75 kDa), carbonic anhydrase (29 kDa), aprotinin (6.5 kDa) ovalbumin (43 kDa), and RNase (13.7 kDa).

### Diffusion plate assay.

Diffusion assays ware carried out as previously described with modification (100). Briefly, exponentially growing C. difficile R20291 cells were harvested and washed twice with 50 mM Tris-HCl (pH 7.4). An agar powder (7.5 g/l) was added to resuspended cells (OD_600_ = 2) and autoclaved at 121°C for 15 min. One milliliter of solution was added into individual well of a 6-well plate (Nunc). After solidification, a 7-mm diameter was created at the center of each well. Next, 15 μL of 100 μM the 6×His-CD16/50L^(WT)^, 6×His-CD16/50L^(W257A)^, and 6×His-EAD endolysin variants were applied into the hole and incubated at 37°C for overnight. Diffusion was evaluated by measuring the diameter of clear zone around each hole.
