# Supplementary material for: Potential Role of the Host-Derived Cell-Wall Binding Domain of Endolysin CD16/50L as a Molecular Anchor in Preservation of Uninfected Clostridioides difficile for New Rounds of Phage Infection
Source: Microbiol Spectr. 2022 Apr 4;10(2):e02361-21. doi: 10.1128/spectrum.02361-21 (PMC9045149; doi:10.1128/spectrum.02361-21)
Supplement: SUPPLEMENTAL FILE 1 — Supplemental material. Download SPECTRUM02361-21_Supp_1_seq8.pdf, PDF file, 5.4 MB [file spectrum02361-21_supp_1_seq8.pdf]

Supplementary information

Potential role of the host-derived cell-wall binding domain of endolysin CD16/50L as a molecular anchor in preservation of uninfected *Clostridioides difficile* for new infection rounds

Wichuda Phothichaisri, Jirayu Nuadthaisong, Tanaporn Phetruen, Tavan Janvilisri, Robert Fagan, Surang Chankhamhaengdecha, Sittinan Chanarat

Supplementary information includes:

Figures S1-S5

Tables S1-S6

Supplemental references

Figure S1

A

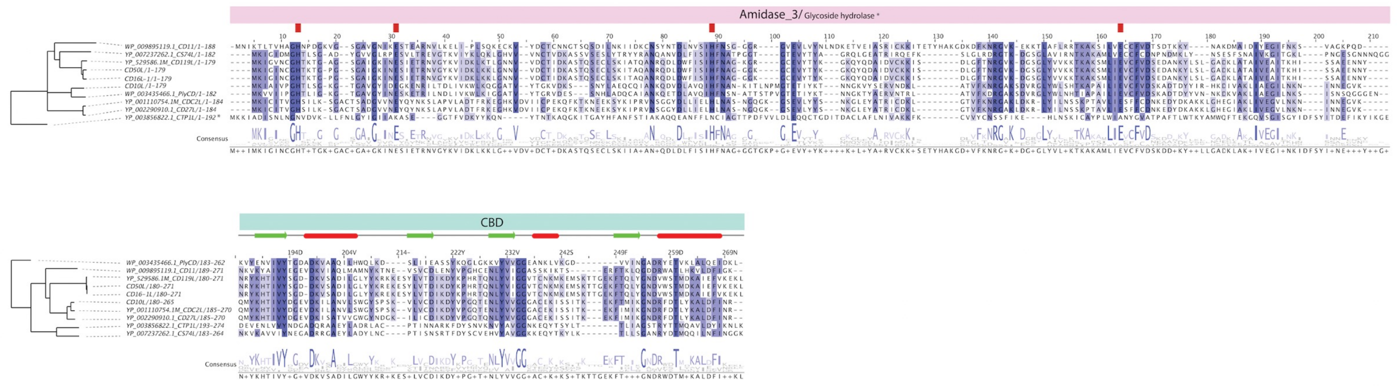

B

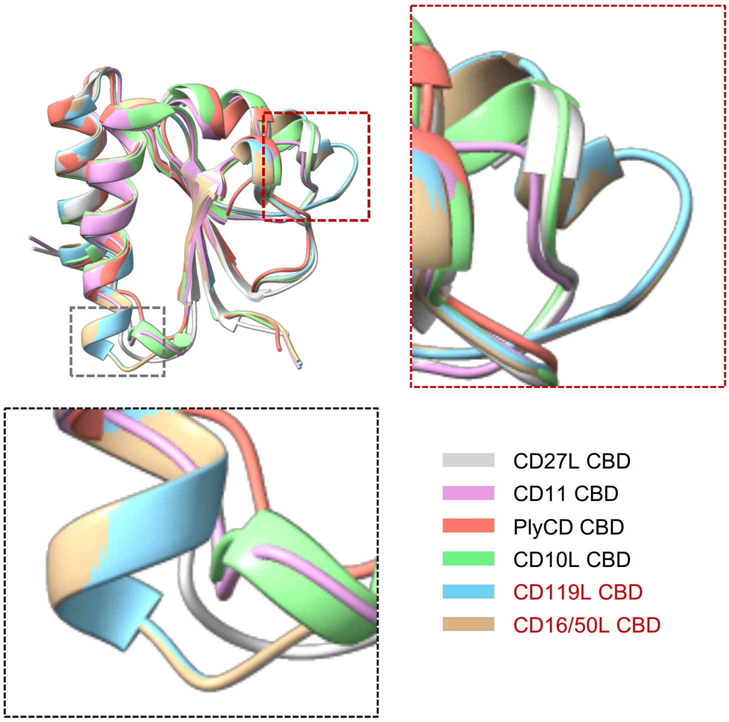

C

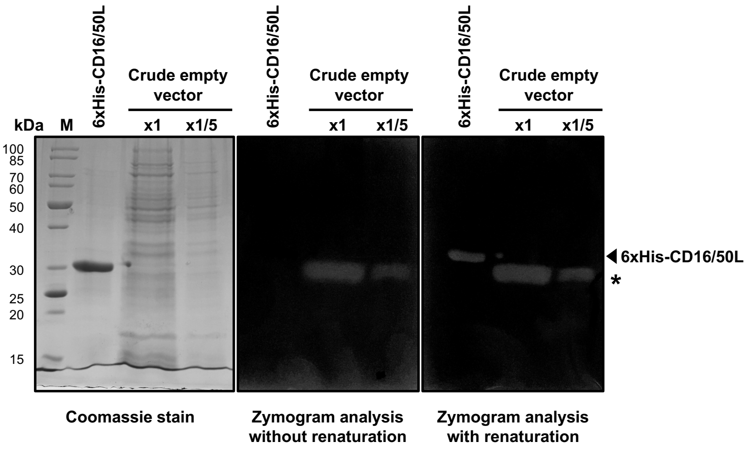

Figure S2

A

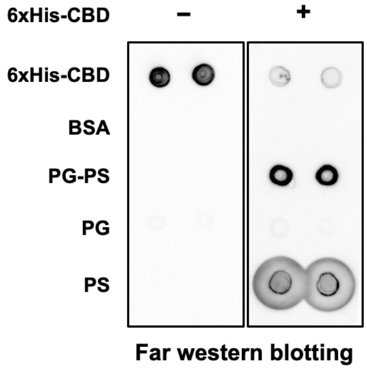

B

Shine-dalgarno sequence — Alternative start codon

Endolysin																
CTP1L	550	GAA	UUU	AUA	AAA	UAU	AUU	AAG	GGG	GAA	GAU	GAA	GUG	GAA	AAU	UUA
	184	E	F	I	K	Y	I	K	G	E	D	E	V	E	N	L
CS74L	520	GAA	UCU	GGA	AAC	AAU	AAU	CAA	GGG	GGU	AAU	AAA	GUG	AAA	GCA	GUA
	174	E	S	G	N	N	N	Q	G	G	N	K	V	K	A	V
CD27L	523	AAU	AAA	AAU	AUA	AAU	AAU	GAG	GGA	GUU	AAA	CAG	AUG	UAC	AAA	CAU
	175	N	K	N	I	N	N	E	G	V	K	Q	M	Y	K	H
CD10L	508	AAU	AAA	AAU	AUA	AAU	AAU	GAG	GGA	GUU	AAA	CAG	AUG	UAC	AAA	CAU
	170	N	K	N	I	N	N	E	G	V	K	Q	M	Y	K	H
CD16/50L	505	AAA	CAU	AUA	AGU	UCA	GCA	GAA	GAA	AAC	AAU	UAU	AAU	AGA	UAU	AAA
	169	K	H	I	S	S	A	E	E	N	N	Y	N	R	Y	K

Endolysin mRNA/amino acid

Figure S3

A

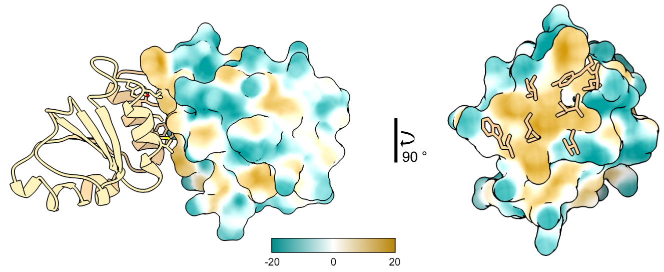
90 °
-20
0
20

B

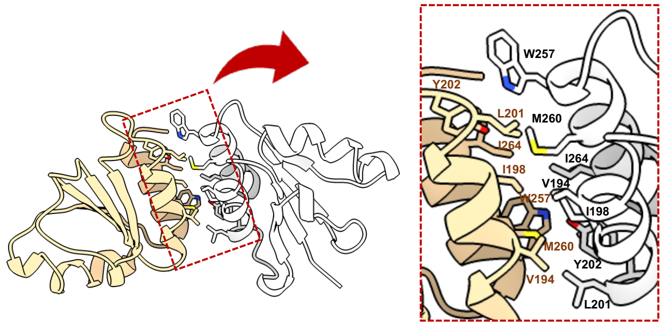
W257
Y202
L201
M260
I264
I264
I198
V194
W257
I198
M260
Y202
V194
L201

Figure S4

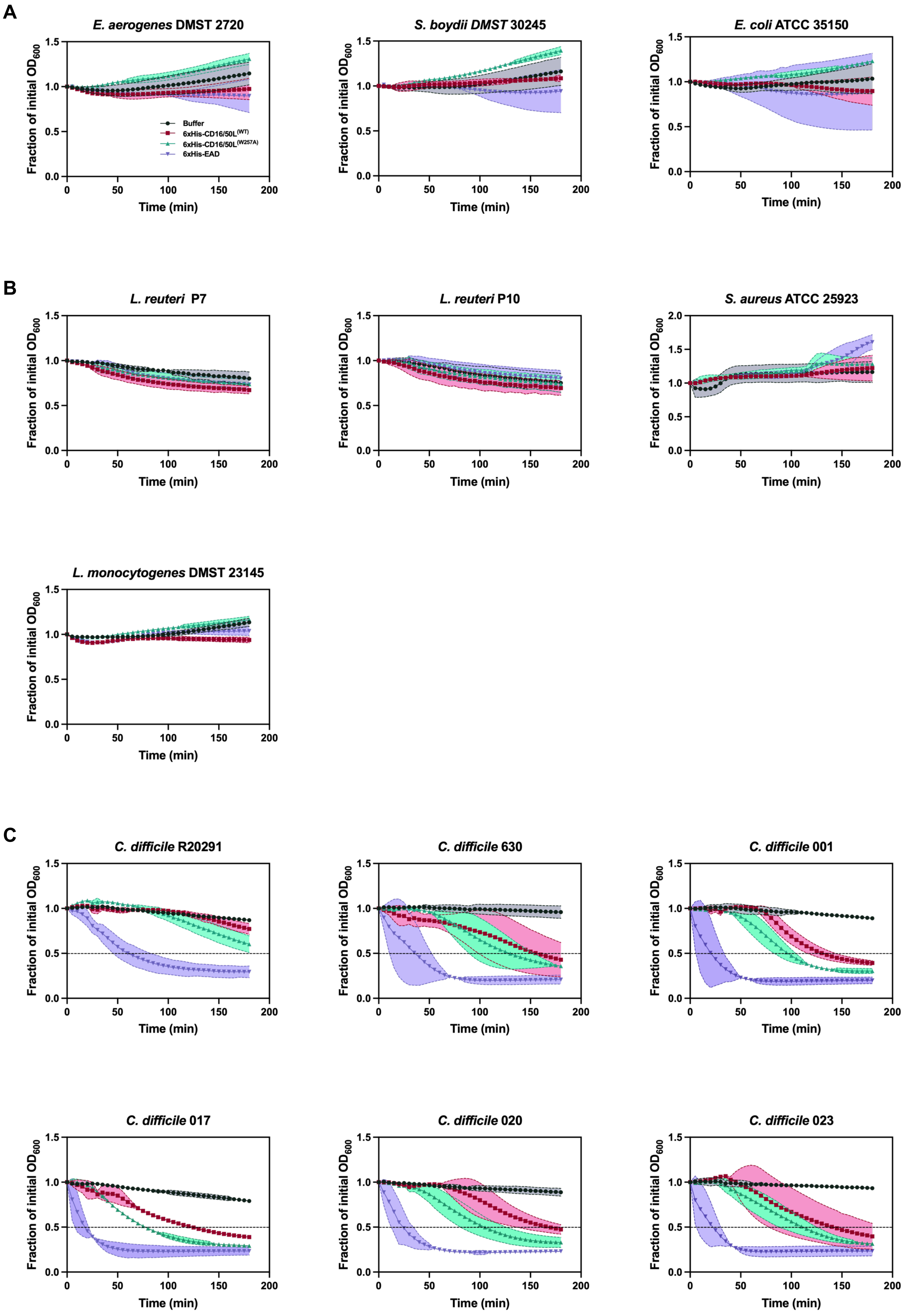

A
E. aerogenes DMST 2720
S. boydii DMST 30245
E. coli ATCC 35150
Buffer
6xHis-CD16/50L(WT)
6xHis-CD16/50L(W257A)
6xHis-EAD
B
L. reuteri P7
L. reuteri P10
S. aureus ATCC 25923
L. monocytogenes DMST 23145
C
C. difficile R20291
C. difficile 630
C. difficile 001
C. difficile 017
C. difficile 020
C. difficile 023
Fraction of initial OD600
Time (min)

Figure S4 (cont.)

C (cont.)

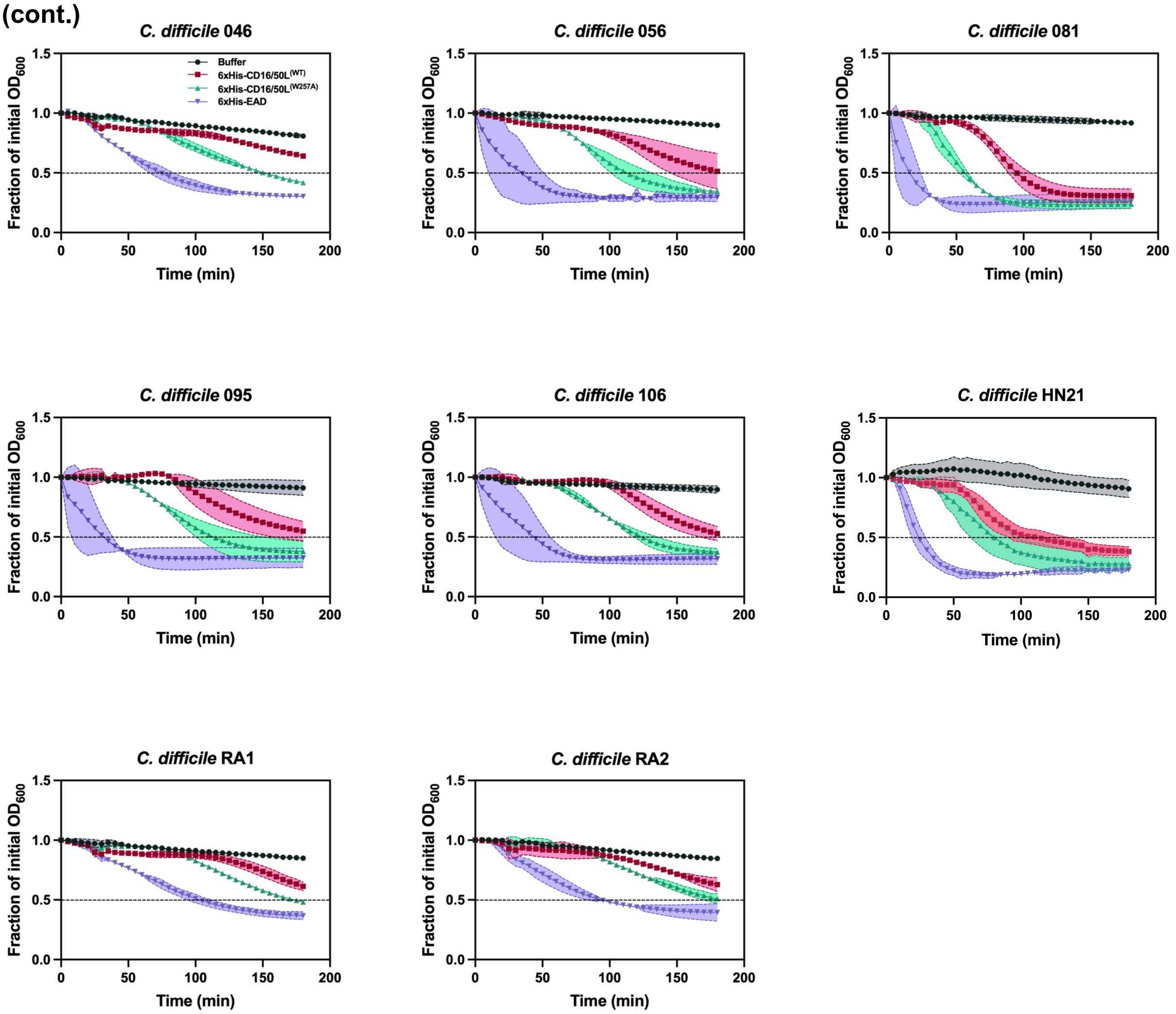

D

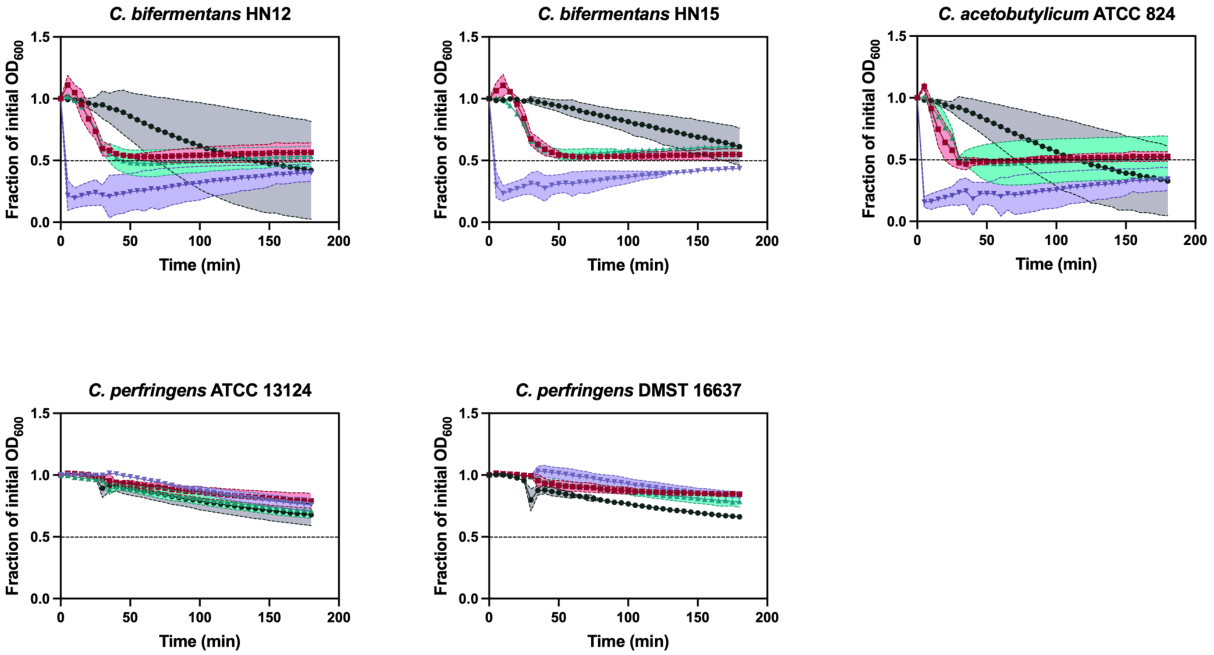

Figure S5

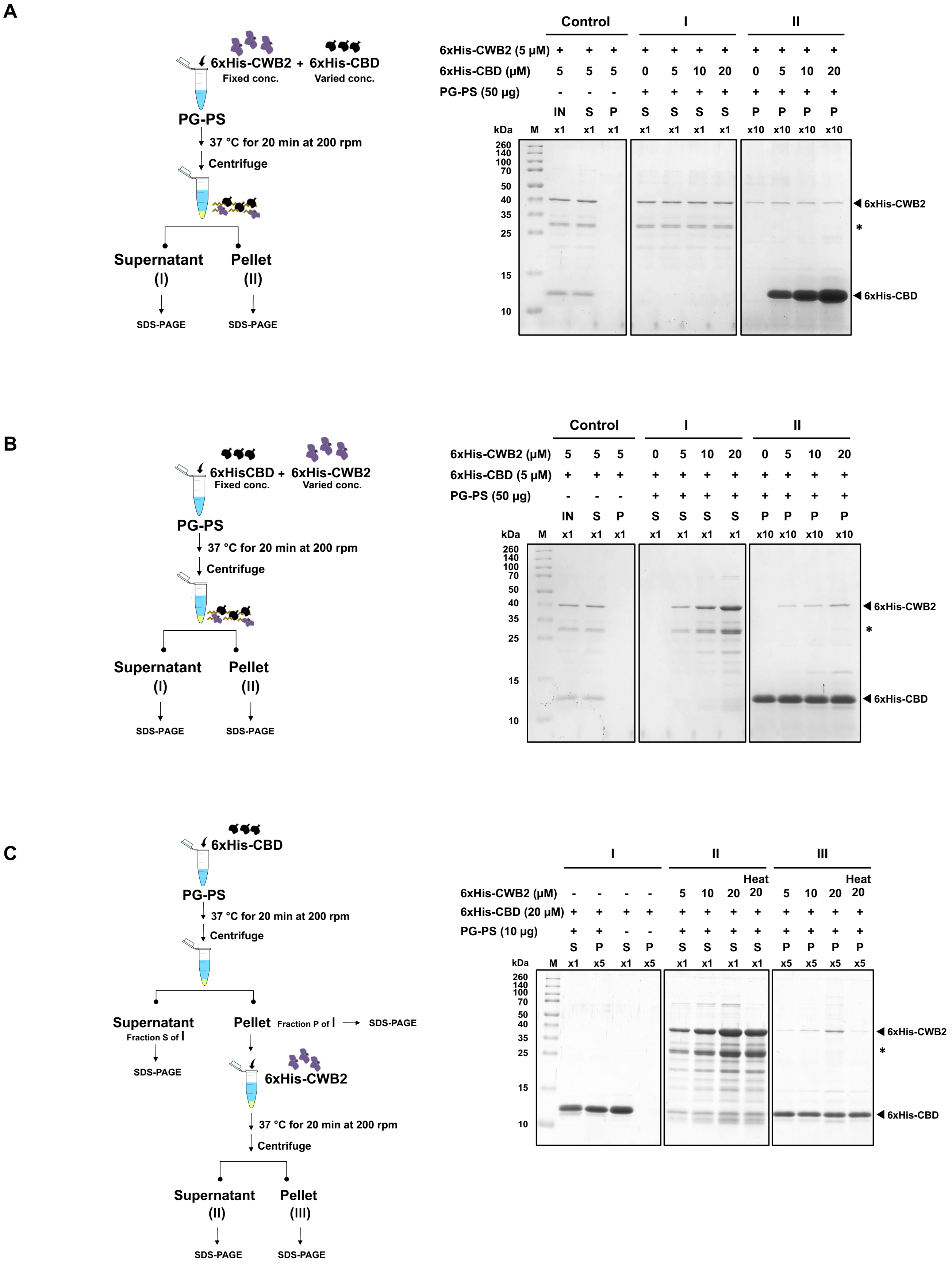

A
6xHis-CWB2 + 6xHis-CBD
Fixed conc.
Varied conc.
PG-PS
37 °C for 20 min at 200 rpm
Centrifuge
Supernatant (I)
Pellet (II)
SDS-PAGE
Control
I
II
6xHis-CWB2 (5 µM)
6xHis-CBD (µM)
PG-PS (50 µg)
IN S P
x1 x10
kDa
M
260 140 100 70 50 40 35 25 15 10
6xHis-CWB2
*
6xHis-CBD
B
6xHisCBD + 6xHis-CWB2
Fixed conc.
Varied conc.
6xHis-CWB2 (µM)
6xHis-CBD (5 µM)
C
6xHis-CBD
Supernatant
Fraction S of I
Pellet
Fraction P of I
6xHis-CWB2
Supernatant (II)
Pellet (III)
III
Heat
6xHis-CBD (20 µM)
PG-PS (10 µg)
x5

SUPPLEMENTARY FIGURE LEGENDS

FIG S1 The CD16/50L is distinct from well-characterized endolysins of *C. difficile* phages

(A) Multiple sequence alignment of CD10L, CD16-1L, CD50L and other endolysins isolated from phages infecting *C. difficile, C. tyrobutyricum, and C. sordellii.* Sequences of EAD (top panel) and CBD (bottom panel) were aligned separately by Clustal Omega (version 1.2.4) (1). Jalview was used to virtualize the results (version 2.11.1.4) (2). The aligned amino acid sequences, conservation scores, and the consensus sequences are shown.

(B) The CD119L and CD16/50L CBD displayed two extended loops distinct from related *C. difficile* phage endolysins. The CBD structure models of CD11, PlyCD, CD119L, CD10L and CD16/50L were predicted using AlphaFold (3). Protein structure comparison and visualization were performed by using the UCSF ChimeraX-daily version 1.3 (2021-09-07) (4). Insets show the magnified images of the extended loop regions.

(C) A false positive result in zymogram assay. A five-fold dilution of crude lysate from *E. coli* BL21 (DE3) was resolved by SDS-PAGE followed by Coomassie blue stain (left) and by zymogram analysis using of *C. difficile* cells as peptidoglycan substrate (middle and right). The purified CD16/50L serves as a positive control. The SDS gel containing *C. difficile* peptidoglycan was analyzed in an absence (middle) or presence (right) of renaturation treatment. Asterisk indicates a highly positively charged protein band showing a false positive result.

FIG S2 CD16/50L CBD interacts with the polysaccharide type II (PS-II) and its characteristics is distinct from reported endolysins.

(A) CBD of CD16/50L binds to the PS-II component of *C. difficile* cell wall**.** Analysis similar to Fig. 2D, but the cell-wall components of *C. difficile* strain 630 was used.

(B) Scheme of an alternative start codon of the endolysins of phages infecting clostridial species. The mRNA sequence shows the upstream region of the endolysin CBD. The Shine-Dalgarno sequence is indicated in red, and the predicted alternative start codon underlined. Amino acid is denoted relative to respective mRNA sequence. Numbers indicate nucleotide or amino acid positions.

FIG S3 The dimerization of CD16/50 CBD confers by a hydrophobic interaction

(A) The dimerization of CD16/50 CBD is most likely via a hydrophobic interaction. The homo-dimeric conformation of CBD is shown as ribbon and hydrophobic surface models (left panel). Hydrophobicity of surface areas was calculated and displayed as a spectrum from cyan (hydrophilic) to goldenrod (hydrophobic). The hydrophobic residues located at the dimer interface are shown as a stick model. Two views of the structures are related by a 90-degree rotation.

(B) The dimer interface of CD16/50 CBD is enriched with hydrophobic amino acid residues. Ribbon model shows the homodimeric structure model of the CD16/50L CBD. Close-up of the dimer interface is shown in the red inset. The hydrophobic residues located at the dimer interface are labeled.

FIG S4 A range of cytolytic activity of CD16/50L.

(A) Similar to Fig. 1C, but CD16/50L protein variants were analyzed against Gram-negative bacterial cells.

(B) Similar to Fig. 1C, but CD16/50L protein variants were analyzed against Gram-positive bacterial cells.

(C) Similar to Fig. 1C, but CD16/50L protein variants were analyzed against different strains of *C. difficile.*

(D) Similar to Fig. 1C, but CD16/50L protein variants were analyzed against relative *Clostridial* species.

Mean ± SD are shown (n = 2).

FIG S5 Competitive binding study of the CD16/50L CBD and CWB2 to PG-PS complex.

(A) (Left panel) A diagram depicts a competitive co-incubation assay. (Right panel) Fifty µg of the purified PG-PS complex was mixed with indicated concentrations of 6xHis-CBD and 6xHis-CWB2. The mixtures were incubated at 37°C for 20 min with agitation rate of 200 rpm. The PG-PS bound proteins were precipitated by centrifugation, resulting a supernatant (I) and pellet (II) fractions. The pellets were washed and resuspended in a one-tenth of the original volume (x10). Then an equal volume of input (IN), supernatant (S), and pellet (P) were subjected to SDS-PAGE, followed by Coomassie blue stain. The 6xHis-CWB2 and 6xHis-CBD positions are marked as arrowhead. The asterisk denotes a degraded fragment of the purified protein.

(B) Similar to Fig. S5A, but the concentration of 6xHis-CBD was set constant at 5 µM and 6xHis-CWB2 concentrations were varied as indicated.

(C) (Left) A diagram depicts a competitive displacement assay. (Right) First, 10 µg of purified PG-PS complex was mixed with 50 µl of 20 µM of 6xHis-CBD and incubated at 37°C for 20 min. After centrifugation, supernatant and part of pellet fraction (I) were collected for SDS-PAGE analysis. After a

washing step, the PG-PS bound pellets were treated with purified native or heat-treated 6xHis-CBD (serving as control) at indicated concentrations. The mixtures were incubated at 37 °C for 20 min and then precipitated by centrifugation, resulting supernatant (II) and pellet fractions. The pellets were washed and resuspended in a one-fifth of the original volume (x5) (III). Then equal volumes of input (IN), supernatant (S), and pellet (P) were subjected to SDS-PAGE, followed by Coomassie blue stain.

Table S1 CBD is necessary for hydrolytic activity of certain, but not all endolysins. It has been demonstrated that removing of CBD from endolysin enhanced cytolytic activity. While the activity of some endolysins was reduced or abolished when the CBD was deleted.

Table S2 The CBD of *C. difficile* phage endolysins shows the secondary structure similarity to CWB2 domain. Sequence similarity searching was performed using HHpred (5). The CBD sequences from CD16/50L and CD27L were used as a query for protein remote homology detection. The *C. difficil*e cell-wall proteins such as Cwp8, Cwp6, Cwp11, and Cwp29 in addition to endolysins were detected as homologous proteins with high probability score from 72% to 93% to be true positives. Based on Hidden Markov model, the aligned region of the protein templates was identified as the CWB2 domain, a cell-wall binding domain 2 (Pfam: PF04122) of *C. difficile* surface and cell-wall proteins.

Table S3 The CBD of phage endolysins is likely to derived from their bacterial host proteins. The CBD of endolysin was analyzed against their respective host protein using Pfam and UniProt database.

Table S4 Strains used in this study.
Table S5 Plasmids used in this study.
Table S6 Primers used in this study.

Table S1 CBD is necessary for hydrolytic activity of certain, but not all endolysins.

No.	Bacterial host	Phage/Protein	Endolysin	Enzymatically active domains [b]	Cell wall binding domain	Lytic activity [a]		Reference
						FL	EAD	
1	*Clostridium tyrobutyricum*	*phiCTP1*	CTP1L	Glycosyl hydrolases family 25	CWB2	+++	-	(6)
2	*Bacillus cereus*	*phage PBC5*	LysPBC5	Glycosyl hydrolases family 25	SH3_5	+++	+	(7)
3	*Bacillus anthracis*	*Phage BcpI*	PlyB	Glycosyl hydrolases family 25	SH3_5	+++	-	(8)
4	*Streptococcus pneumoniae*	*Phage Cp-7*	CpL-7	Glycosyl hydrolases family 25	CW_7	+++	+	(9)
5	*Listeria monocytogenes*	*Listeria phage P40*	PlyP40	Glycosyl hydrolases family 25	LysM and SH3_3	+++	-	(10)
6	*Clostridioides difficile*	*phiNH16-1*and *phiHN50*	CD16/50L	Amidase_3	CWB2	+	+++	This study
7	*C. difficile*	*phiCD27*	CD27L	Amidase_3	CWB2	+	+++	(11)
8	*C. difficile*	cell wall hydrolase CD630	PlyCD	Amidase_3	CWB2	+	+++	(12)
9	*C. difficile*	Autolysin	CD11	Amidase_3	CWB2	+	+++	(13)
10	*B. anthracis*	*lambda-like prophage Ba02 endolysin*	PlyL	Amidase_2	CBD_PlyG	+	+++	(14)
11	*L. monocytogenes*	*Listeria phage PSA*	PlyPSA	Amidase_3	CBD_PSA	+++	+	(15)
12	*L. monocytogenes*	*Listeria phage A511*	Ply511	Amidase_2	DUF3597	+	+++	(16)
13	*Streptococcus sp.*	*streptococcal bacteriophage C1*	PlyC	Endopeptidase (CHAP)	PlyCB	+++	-	(17)
14	*L. monocytogenes*	*Listeria phage A118*	Ply118	Endopeptidase (L-alanyl-D-glutamate peptidase)	DUF3597	+++	-	(18)
15	*L. monocytogenes*	*Listeria phage A500*	Ply500	Endopeptidase (L-alanyl-D-glutamate peptidase)	CBD_PSA	+++	-	(18)
16	*Staphylococcus aureus*	*Staphylococcal phage SA12*	LysSA12	Endopeptidase (CHAP)-Amidase_2	SH3_5	+++	CHAP + Amidase -	(19)
17	*S. aureus*	*Staphylococcal phage SA97*	LysSA97	Endopeptidase (CHAP)-Amidase_3	SH3_5	+++	CHAP + Amidase -	(19)
18	*S. dysgalactiae*	*S. dysgalactiae* SK1249	PlySK1249	Amidase-Endopeptidase (CHAP)	LysM	+++	Amidase + Amidase_LysM ++ CHAP_LysM -	(20)

[a] FL, full-length; EAD, enzymatically active domain; Cytolytic activity: +++, high; +, low; -, no.

[b]CHAP, a cysteine, histidine-dependent amidohydrolases/peptidase

Table S2 The CBD of *C. difficile* phage endolysins shows the secondary structure similarity to CWB2 domain.

No.	Accession No.	Organism	Protein	Protein domain	Propability[a]	E-value	Identity[b]	Query HMM region	Template
	**Query: CD16/50L CBD sequence**								
1	4CU5_B	*C. difficile phage*	CD27L	CBD	99.8%	1.4×10^{-17}	87%	2-90	1-85
2	5A6S_B	*C. tyrobutyricum phage*	CTP1L	CBD	99.5%	5.5×10^{-13}	24%	3-91	1-79
3	5J72_A	*C. difficile*	Cwp6	CWB2	72.1%	28	8%	40-88	102-151
4	2V5C_A	*C. perfringens*	Glycosidase	Glycosidase	45%	200	13%	6-89	37-131
5	d5nula_c	*C. beijerinckii*	Flavodoxin	Flavodoxin	43.2%	90	13%	6-53	2-54
	**Query: CD27L CBD sequence**								
1	5A6S_B	*C. tyrobutyricum phage*	CTP1L	CBD	99.6%	3.6×10^{-15}	22%	7-89	1-79
2	WP_003435466.1	*C. difficile*	Amidase	CBD	98.6%	9.3×10^{-12}	34%	1-90	179-261
3	WP_021364727.1	*C. difficile*	Cwp11	CWB2	93.7%	1.8	22%	15-88	162-235
4	WP_021373596.1	*C. difficile*	Cwp29	CWB2	91.3%	5.7	23%	14-88	160-234
5	WP_003438171.1	*C. difficile*	Cwp26	CWB2	90.8%	4.9	21%	14-88	161-235
6	WP_021369084.1	*C. difficile*	Cwp28	CWB2	87.1%	18	24%	14-88	173-247
7	5J72_A	*C. difficile*	Cwp6	CWB2	85.4%	7.7	8%	8-88	56-151
8	WP_021359059.1	*C. difficile*	Amidase	CWB2	84.2%	33	19%	8-88	191-286
9	WP_004454101.1	*C. difficile*	Cwp5	CWB2	84.1%	32	19%	15-88	165-246
10	5J6Q_A	*C. difficile*	Cwp8	CWB2	80.3%	36	21%	9-88	420-509

[a] Probability of the template being a true positive.

[b] Percent sequence identity between the template and query sequences.

HMM, hidden Markov model; CBD, Cell wall binding domain; CWB2, Cell wall binding domain_2.

Table S3 The CBD of phage endolysins is likely to derived from their bacterial host proteins.

Bacterial host	Endolysin/gene	Accession no.	Phage/protein	Cell-wall binding domain											
				CWB2: PF04122	SLH: PF00395	CB: PF014473, PF19127	PG binding: PF01471	LysM: PF01476	SH3b: PF08239, PF08460	CW_7: PF08230	PAS CBD: PF18341	CBD_PlyG: PF12123	DUF3597: PF12200	DUF5776: PF19087	PlyCB[a]
Clostridioides difficile	CD27L	B6SBV8	*phiCD27*	◩		■	■	■	■						
C. difficile	CD11	WP_009895119.1	Cell wall hydrolase CD630	◩		■	■	■	■						
C. difficile	PlyCD	Q187L1	Cell wall hydrolase CD630	◩		■	■	■	■						
C. difficile	CD2L	A3QSC7	*phiC2*	◩		■	■	■	■						
C. difficile	CD119L	Q24LG3	*phiCD119*	◩		■	■	■	■						
Clostridium sporogenes	CS74L	I1TJX3	*Clostridium phage phi5074-B1*	◩			■	■	■						
Clostridium tyrobutyricum	CTP1L	D9ZNF3	*phiCTP1*	◩			■	■	■						
Clostridium botulinum	CBO1751	A5I2M1	*Prophage CB*	■	■	■	■	■	◩						
Clostridium perfringens	Ply3626	Q8SBN4	*Clostridium phage phi3626*	■		■	■	■	◩						
C. perfringens	PSm	Q0SPG7	*Clostridium phage phiSM101*	■		■	■	■	◩						
C. perfringens	phiCP51L	M9Q1I7	*Clostridium phage vB_CpeS-CP51*	■		■	■	■	◩						
Bacillus subtilis	phi29L	P11187	*Bacillus phage phi29*	■		■	■	◩	■						
Bacillus cereus	PysBPS13	J9PU17	*Bacillus phage BPS13*	■		■	■	■	◩	■					
B. cereus	LysB4	H9NAL3	*Bacillus phage B4*	■		■	■	■	◩	■					
B. cereus	LysPBC2	A0A218KC88	*Bacillus phage PBC2*	■		■	■	■	◩	■					
B. cereus	LysPBC5	A0A218KCJ1	*Bacillus phage PBC5*		■	■	■	■	◩	■					
B. cereus	Ply21	P89924	*Bacillus phage TP21-L*		■	■	■	■	◩	■					
Bacillus anthracis	PlyL	AAP27798	*lambda-like prophage Ba02*		■	■	■	■	■			◩			
B. anthracis	PlyG	Q8LTE6	*Bacillus grama phage*		■	■	■	■	■			◩			
B. anthracis	PlyB	X2JN49	*Bacteriophage vB_BanS_Bcp1*		■	■	■	■	◩						
Staphylococcus aureus	LysSA12	S4V6E6	*Staphylococcal phage SA12*			■	■	■	◩						
S. aureus	LysSA97	A0A0F6N3N1	*Staphylococcal phage SA97*			■	■	■	◩						
S. aureus	LysK	Q6Y7T6	*Staphylococcus phage K*			■	■	■	◩						
Listeria monocytogenes	Ply511	Q38653	*L. monocytogenes phage A511*				■	■	■				◩		
L. monocytogenes	Ply118	Q37976	*L. monocytogenes phage A118*				■	■	■					◩	
L. monocytogenes	Ply500	Q37979	*L. monocytogenes phage A500*				■	■	■		◩				
L. monocytogenes	PlyPSA	Q8W5Y8	*Phage PSA*				■	■	■		◩				
L. monocytogenes	PlyP35	6THJ	*L. monocytogenes phage P35*				■	■	■		◩				
L. monocytogenes	PlyP40	B6D7J9	*L. monocytogenes phage P40*				■	◩	◩						
Streptococcus sp.	PlyC	Q7Y3F1, Q7Y3F3	*streptococcus phage C1*			■	■	■	■						◩
Streptococcus agalactiae	phage 30B lysin	Q8HA43	*Streptococcus phage B30*				■	■	◩						
Streptococcus pneumoniae	CpL-1	P15057	*Streptococcus phage CP-1*			◩	■	■	■						
S. pneumoniae	CpL-7	P19385	*Streptococcus phage Cp-7*			■	■	■	■	◩					
Enterococcus sp.	N/A	Q6E4B1	*Enterococcus sp. Phage 1*		■	■	■	■	◩						
Enterococcus faecalis	PEf771_50	A0A5C2H0F2	*Enterococcus phage PEf771*		■	■	■	■	◩						

■ Domains that are present in host genomes

◩ Domains that are present in both genomes of bacterial host and phage

[a] Unassigned domain in Pfam database

CWB2, Cell wall binding domian_2; SLH, S-layer homology domain; CB, Choline-binding repeat; PG_binding, Peptidoglycan binding domain; LysM, Lysin motif domain; SH3b, Bacterial src-homology 3 domain; CW_7, Cell wall repeat_7; PAS CBD, PSA endolysin cell wall binding domain; CBD_PlyG, PlyG cell wall binding domain; DUF, Domain of unknown function; PlyCB, PlyCB cell wall binding domain

Table S4 Strains used in this study.

**Strains/Ribotypes**	**Reference**
Clostridiodes difficile FM2.5 (R20291 S-layer mutant)	(21)
C. difficile R20291	N. Minton, UK
C. difficile 630	N. Minton, UK
C. difficile 001	N. Minton, UK
C. difficile 017	N. Minton, UK
C. difficile 020	N. Minton, UK
C. difficile 023	N. Minton, UK
C. difficile 046	N. Minton, UK
C. difficile 056	N. Minton, UK
C. difficile 081	N. Minton, UK
C. difficile 095	N. Minton, UK
C. difficile 106	N. Minton, UK
C. difficile HN21	(22)
C. difficile RA1	(22)
C. difficile RA2	(22)
Escherichia coli BL21 (DE3)	Novagen
E. coli Rosetta (DE3)	Novagen
E. coli XL10-Gold	Agilent
E. coli NEB 5α	New England Biolabs
E. coli CA434 (HB101 carrying R702)	R. Fagan, UK
E. coli O157:H7 ATCC 35150	ATCC
Clostridium bifermentans HN12	(22)
C. bifermentans HN15	(22)
Clostridium acetobutylicum ATCC 824	ATCC
Clostridium perfringens ATCC 13124	ATCC
C. perfringens DMST 16637	DMST
Lactobacillus reuteri P7	(23)
L. reuteri P10	(23)
Listeria monocytogenes DMST 23145	DMST
Staphylococcus aureus ATCC 25923	ATCC
Enterobacter aerogenes DMST 2720	DMST
Shigella boydii DMST 30245	DMST

ATCC, American Type Culture Collection, USA; DMST, Department of Medical Sciences Thailand, Thailand

Table S5 Plasmids used in this study.

Plasmid	Genotype [a]	Reference
pWP017	*lacI* P_{T7} *CD16/50L*$_{full\text{-}length}$; *amp*	This study
pWP018	*lacI* P_{T7} *CD16/50L*$_{EAD}$; *amp*	This study
pWP019	*lacI* P_{T7} *CD16/50L*$_{CBD}$; *amp*	This study
pWP024	*lacI* P_{T7} *CD16/50L*$^{(W257A)}$; *amp*	This study
pWP022	*lacI* P_{T7} *CD16/50L*$_{CBD}^{(W257A)}$; *amp*	This study
pWP023	*lacI* P_{T7} *CD16/50L*$_{CBD}^{(Y202,\ W257A)}$; *amp*	This study
pWP020	*lacI* P_{T7} *cwp8*$_{290\text{-}600}$; *amp*	This study
pWP021	*lacI* P_{T7} *mCherry-CBD; km*	This study
pJAK175	*xylR* P_{xyl} *-bitluc*^{opt}; *catP*	JA. Kirk, UK
pAF256	*tetR* P_{tet} *-hupA-smbit/lgbit; catP*	(24)
pAF257	*tetR* P_{tet} *-smbit/hupA-lgbit; catP*	(24)
pAP118	*tetR* P_{tet} *-hupA-smbit/hupA-lgbit; catP*	(24)
pWP001	*xylR* P_{xyl} *-CBD-smbit/-lgbit*; *catP*	This study
pWP002	*xylR* P_{xyl} *-CBD-smbit/hupA-lgbit*; *catP*	This study
pWP003	*xylR* P_{xyl} *-smbit/CBD-lgbit*; *catP*	This study
pWP004	*xylR* P_{xyl} *-CBD-smbit/CBD-lgbit*; *catP*	This study
pWP005	*xylR* P_{xyl} *-CBD*$^{(Y202A)}$*-smbit/CBD*$^{(Y202A)}$*-lgbit*; *catP*	This study
pWP006	*xylR* P_{xyl} *-CBD*$^{(C234A)}$*-smbit/CBD*$^{(C234A)}$*-lgbit*; *catP*	This study
pWP007	*xylR* P_{xyl} *-CBD*$^{(E238A)}$*-smbit/CBD*$^{(E238A)}$*-lgbit*; *catP*	This study
pWP008	*xylR* P_{xyl} *-CBD*$^{(E248A)}$*-smbit/CBD*$^{(F248A)}$*-lgbit*; *catP*	This study
pWP009	*xylR* P_{xyl} *-CBD*$^{(Q250A)}$*-smbit/CBD*$^{(Q250A)}$*-lgbit*; *catP*	This study
pWP010	*xylR* P_{xyl} *-CBD*$^{(W257A)}$*-smbit/CBD*$^{(W257A)}$*-lgbit*; *catP*	This study
pWP011	*xylR* P_{xyl} *-CBD*$^{(M260A)}$*-smbit/CBD*$^{(M260A)}$*-lgbit*; *catP*	This study
pWP016	*xylR* P_{xyl} *-CBD*$^{(Y202A,\ W257A)}$*-smbit/CBD*$^{(Y202A,\ W257A)}$*-lgbit*; *catP*	This study

[a] amp, ampicillin resistance cassette; catP, chloramphenicol resistance cassette; km, kanamycin resistance cassette; *lacI*, *lac* repressor; *tetR*, tet repressor; *xylR*, xylose repressor; P_{T7}, T7 promoter; P_{xyl}, xylose-inducible promoter; P_{tet}, anhydrotetracycline-inducible promoter

Table S6 Primers used in this study.

Primers	Sequence (5'→3') [a]	Features
CD16/50$_{full\text{-}length}$ forward	GACGGTAGCCATATGAAAATAGGTATAAATTGTGGACAT	*Nde*I
CD16/50$_{full\text{-}length}$ reverse	AGCGTTGACCTCGAGTCACAATTTTTCTTTTACAAATTCTA	*Xho*I
CD16/50$_{EAD}$ reverse	GCTCTCGAGTCAATAATTGTTTTCTTCTGCTGAACT	*Xho*I
CD16/50$_{CBD}$ forward	TCGTCCAACCATATGAATAGATATAAACATACAATAGTG	*Nde*I
Cwp8$_{290\text{-}600}$ forward	*ATGCTCGAGGATCCG*GGCAGCGGTTCTGGCTCCGGTAAAGTAGAAGTTTTATCTGGTGATTCA	Homology arm
Cwp8$_{290\text{-}600}$ reverse	*CTCAGCTTCCTTTCG*TTAGTTCTTAGTAAATAAGTTTATTTTTTC	Homology arm
pET15b forward	*CGAAAGGAAGCTGAG*TTGGCT	Homology arm
pET15b reverse	*CGGATCCTCGAGCAT*ATGGCT	Homology arm
CBD$^{(Y202A)}$ forward	TTTAGGACTAgcaTATAAGAGAGAAAAAGAAAGTTACTTAG	Site-directed mutagenesis
CBD$^{(Y202A)}$ reverse	ATGTCTGCTGATACTTTATC	Site-directed mutagenesis
CBD$^{(W257A)}$ forward	TAATGATGTAgcaTCAACAATGGATAAAGCTATAG	Site-directed mutagenesis
CBD$^{(W257A)}$ reverse	CCATATAGTTGAGTAAATTTTTC	Site-directed mutagenesis
CBD$^{(M260A)}$ forward	ATGGTCAACAgcaGATAAAGCTATAGAATTTG	Site-directed mutagenesis
CBD$^{(M260A)}$ reverse	ACATCATTACCATATAGTTGAG	Site-directed mutagenesis
CBD$^{(C234A)}$ forward	TGGAGTAACTgcaAATAAAAATGAAGGAAATGAG	Site-directed mutagenesis
CBD$^{(C234A)}$ reverse	CCAATTACGTACAAATTTTG	Site-directed mutagenesis
CBD$^{(E239A)}$ forward	TAAAATGAAGgcaATGAGTAAGACTAC	Site-directed mutagenesis
CBD$^{(E239A)}$ reverse	TTACAAGTTACTCCACCAATTAC	Site-directed mutagenesis
CBD$^{(F248A)}$ forward	AGGAGAAAAAgcaACTCAACTATATGGTAATG	Site-directed mutagenesis
CBD$^{(F248A)}$ reverse	GTAGTCTTACTCATTTCCTTC	Site-directed mutagenesis
CBD$^{(Q250A)}$ forward	AAAATTTACTgcaCTATATGGTAATGATG	Site-directed mutagenesis
CBD$^{(Q250A)}$ reverse	TCTCCTGTAGTCTTACTC	Site-directed mutagenesis
CBD-smbit forward	GATCGAGCTC**AGGAGG**TACTTATATGAATAGATATAAACATACAATAGTGTACAGTG	*Sac*I
CBD-smbit reverse	GATCCTCGAGACAATTTTTCTTTTACAAATTCTATAGCTTTATC	*Xho*I
CBD-lgbit forward	GATCCGATCG**AGGAGG**TACTTATATGAATAGATATAAACATACAATAGTGTACAGTG	*Pvu*I
CBD-lgbit reverse	GATCGCGGCCGCCAATTTTTCTTTTACAAATTCTATAGCTTTATC	*Not*I
CBD-smbit/CBD-lgbit forward	CAGTAACCAATTTGATATTCCTCC	Sequencing primer

[a] Restriction enzyme site is underlined; Ribosome binding site is shown in bold; Homology arm is shown in italics; Mutation point is denoted by a lowercase.

Supplemental references

1. Madeira F, Park Y mi, Lee J, Buso N, Gur T, Madhusoodanan N, Basutkar P, Tivey ARN, Potter SC, Finn RD, Lopez R. 2019. The EMBL-EBI search and sequence analysis tools APIs in 2019. Nucleic Acids Research 47:W636–W641.

2. Waterhouse AM, Procter JB, Martin DMA, Clamp M, Barton GJ. 2009. Jalview Version 2--a multiple sequence alignment editor and analysis workbench. Bioinformatics 25:1189–1191.

3. Jumper J, Evans R, Pritzel A, Green T, Figurnov M, Ronneberger O, Tunyasuvunakool K, Bates R, Žídek A, Potapenko A, Bridgland A, Meyer C, Kohl SAA, Ballard AJ, Cowie A, Romera-Paredes B, Nikolov S, Jain R, Adler J, Back T, Petersen S, Reiman D, Clancy E, Zielinski M, Steinegger M, Pacholska M, Berghammer T, Bodenstein S, Silver D, Vinyals O, Senior AW, Kavukcuoglu K, Kohli P, Hassabis D. 2021. Highly accurate protein structure prediction with AlphaFold. Nature 596:583–589.

4. Pettersen EF, Goddard TD, Huang CC, Meng EC, Couch GS, Croll TI, Morris JH, Ferrin TE. 2021. UCSF ChimeraX : Structure visualization for researchers, educators, and developers. Protein Science 30:70–82.

5. Zimmermann L, Stephens A, Nam S-Z, Rau D, Kübler J, Lozajic M, Gabler F, Söding J, Lupas AN, Alva V. 2018. A Completely Reimplemented MPI Bioinformatics Toolkit with a New HHpred Server at its Core. Journal of Molecular Biology 430:2237–2243.

6. Mayer MJ, Payne J, Gasson MJ, Narbad A. 2010. Genomic Sequence and Characterization of the Virulent Bacteriophage φCTP1 from *Clostridium tyrobutyricum* and Heterologous Expression of Its Endolysin. Appl Environ Microbiol 76:5415–5422.

7. Lee KO, Kong M, Kim I, Bai J, Cha S, Kim B, Ryu K-S, Ryu S, Suh J-Y. 2019. Structural Basis for Cell-Wall Recognition by Bacteriophage PBC5 Endolysin. Structure 27:1355-1365.e4.

8. Porter CJ, Schuch R, Pelzek AJ, Buckle AM, McGowan S, Wilce MCJ, Rossjohn J, Russell R, Nelson D, Fischetti VA, Whisstock JC. 2007. The 1.6 Å Crystal Structure of the Catalytic Domain of PlyB, a Bacteriophage Lysin Active Against Bacillus anthracis. Journal of Molecular Biology 366:540–550.

9. Bustamante N, Iglesias-Bexiga M, Bernardo-García N, Silva-Martín N, García G, Campanero-Rhodes MA, García E, Usón I, Buey RM, García P, Hermoso JA, Bruix M, Menéndez M. 2017. Deciphering how Cpl-7 cell wall-binding repeats recognize the bacterial peptidoglycan. Sci Rep 7:16494.

10. Romero P, Bartual SG, Schmelcher M, Glück C, Hermoso JA, Loessner MJ. 2018. Structural insights into the binding and catalytic mechanisms of the *Listeria monocytogenes* bacteriophage glycosyl hydrolase PlyP40: Structural and functional analyses of PlyP40. Molecular Microbiology 108:128–142.

11. Mayer MJ, Garefalaki V, Spoerl R, Narbad A, Meijers R. 2011. Structure-Based Modification of a Clostridium difficile-Targeting Endolysin Affects Activity and Host Range. J Bacteriol 193:5477–5486.

12. Wang Q, Euler CW, Delaune A, Fischetti VA. 2015. Using a Novel Lysin To Help Control Clostridium difficile Infections. Antimicrob Agents Chemother 59:7447–7457.

13. Wu X, Paskaleva EE, Mehta KK, Dordick JS, Kane RS. 2016. Wall Teichoic Acids Are Involved in the Medium-Induced Loss of Function of the Autolysin CD11 against Clostridium difficile. Sci Rep 6:35616.

14. Low LY, Yang C, Perego M, Osterman A, Liddington R. 2011. Role of Net Charge on Catalytic Domain and Influence of Cell Wall Binding Domain on Bactericidal Activity, Specificity, and Host Range of Phage Lysins. Journal of Biological Chemistry 286:34391–34403.

15. Schmelcher M, Tchang VS, Loessner MJ. 2011. Domain shuffling and module engineering of *Listeria* phage endolysins for enhanced lytic activity and binding affinity: Engineering of Listeria phage endolysins. Microbial Biotechnology 4:651–662.

16. Gaeng S, Scherer S, Neve H, Loessner MJ. 2000. Gene Cloning and Expression and Secretion of *Listeria monocytogenes* Bacteriophage-Lytic Enzymes in *Lactococcus lactis*. Appl Environ Microbiol 66:2951–2958.

17. McGowan S, Buckle AM, Mitchell MS, Hoopes JT, Gallagher DT, Heselpoth RD, Shen Y, Reboul CF, Law RHP, Fischetti VA, Whisstock JC, Nelson DC. 2012. X-ray crystal structure of the streptococcal specific phage lysin PlyC. Proceedings of the National Academy of Sciences 109:12752–12757.

18. Loessner MJ, Kramer K, Ebel F, Scherer S. 2002. C-terminal domains of Listeria monocytogenes bacteriophage murein hydrolases determine specific recognition and high-affinity binding to bacterial cell wall carbohydrates: Cell wall binding domains of Listeria phage lysins. Molecular Microbiology 44:335–349.

19. Son B, Kong M, Ryu S. 2018. The Auxiliary Role of the Amidase Domain in Cell Wall Binding and Exolytic Activity of Staphylococcal Phage Endolysins. Viruses 10:284.

20. Oechslin F, Menzi C, Moreillon P, Resch G. 2021. The multidomain architecture of a bacteriophage endolysin enables intramolecular synergism and regulation of bacterial lysis. Journal of Biological Chemistry 296:100639.

21. Kirk JA, Gebhart D, Buckley AM, Lok S, Scholl D, Douce GR, Govoni GR, Fagan RP. 2017. New class of precision antimicrobials redefines role of *Clostridium difficile* S-layer in virulence and viability. Sci Transl Med 9:eaah6813.

22. Chankhamhaengdecha S, Hadpanus P, Aroonnual A, Ngamwongsatit P, Chotiprasitsakul D, Chongtrakool P, Janvilisri T. 2013. Evaluation of Multiplex PCR with Enhanced Spore Germination for Detection of *Clostridium difficile* from Stool Samples of the Hospitalized Patients. BioMed Research International 2013:1–6.

23. Pipatthana M. 2016. A Bacteriocin derived from Bifidobacterium with high antimicrobial activity against Clostridium difficile. (MSc's thesis)

24. Oliveira Paiva AM, Friggen AH, Qin L, Douwes R, Dame RT, Smits WK. 2019. The Bacterial Chromatin Protein HupA Can Remodel DNA and Associates with the Nucleoid in Clostridium difficile. Journal of Molecular Biology 431:653–672.
